# Specific plasma microRNAs are associated with CD4^+^ T-cell recovery during suppressive antiretroviral therapy for HIV-1

**DOI:** 10.1097/QAD.0000000000003853

**Published:** 2024-02-01

**Authors:** Stefanie Kroeze, Neeltje A. Kootstra, Ad C. van Nuenen, Theresa M. Rossouw, Cissy M. Kityo, Margaret Siwale, Sulaimon Akanmu, Kishor Mandaliya, Marleen de Jager, Pascale Ondoa, Ferdinand W. Wit, Peter Reiss, Tobias F. Rinke de Wit, Raph L. Hamers

**Affiliations:** a Amsterdam Institute for Global Health and Development; b Amsterdam UMC location University of Amsterdam, Department of Global Health; c Amsterdam UMC location University of Amsterdam, Laboratory for Experimental Immunology, Meibergdreef 9; d Amsterdam Institute for Infection and Immunity, Infectious Diseases, Amsterdam, The Netherlands; e Department of Immunology, University of Pretoria, Pretoria, South Africa; f Joint Clinical Research Centre, Kampala, Uganda; g Lusaka Trust Hospital, Lusaka, Zambia; h Department of Haematology and Blood Transfusion, College of Medicine of the University of Lagos and the Lagos University Teaching Hospital, Lagos, Nigeria; i Coast Province General Hospital, Mombasa, Kenya; j Muelmed Hospital, Pretoria, South Africa; k African Society for Laboratory Medicine, Addis Ababa, Ethiopia; l Stichting HIV Monitoring; m Amsterdam UMC location University of Amsterdam, Internal Medicine, Division of Infectious Diseases, Meibergdreef 9, Amsterdam, The Netherlands; n Oxford University Clinical Research Unit Indonesia, Faculty of Medicine Universitas Indonesia, Jakarta, Indonesia; o Nuffield Department of Medicine, Centre for Tropical Medicine and Global Health, University of Oxford, Oxford, UK.

**Keywords:** antiretroviral therapy, CD4^+^ T-cell recovery, HIV-1, immune dysregulation, microRNA

## Abstract

**Objective:**

This study investigated the association of plasma microRNAs before and during antiretroviral therapy (ART) with poor CD4^+^ T-cell recovery during the first year of ART.

**Design:**

MicroRNAs were retrospectively measured in stored plasma samples from people with HIV (PWH) in sub-Saharan Africa who were enrolled in a longitudinal multicountry cohort and who had plasma viral-load less than 50 copies/ml after 12 months of ART.

**Methods:**

First, the levels of 179 microRNAs were screened in a subset of participants from the lowest and highest tertiles of CD4^+^ T-cell recovery (ΔCD4) (*N* = 12 each). Next, 11 discordant microRNAs, were validated in 113 participants (lowest tertile ΔCD4: *n* = 61, highest tertile ΔCD4: *n* = 52). For discordant microRNAs in the validation, a pathway analysis was conducted. Lastly, we compared microRNA levels of PWH to HIV-negative controls.

**Results:**

Poor CD4^+^ T-cell recovery was associated with higher levels of hsa-miR-199a-3p and hsa-miR-200c-3p before ART, and of hsa-miR-17-5p and hsa-miR-501-3p during ART. Signaling by VEGF and MET, and RNA polymerase II transcription pathways were identified as possible targets of hsa-miR-199a-3p, hsa-200c-3p, and hsa-miR-17-5p. Compared with HIV-negative controls, we observed lower hsa-miR-326, hsa-miR-497-5p, and hsa-miR-501-3p levels before and during ART in all PWH, and higher hsa-miR-199a-3p and hsa-miR-200c-3p levels before ART in all PWH, and during ART in PWH with poor CD4^+^ T-cell recovery only.

**Conclusion:**

These findings add to the understanding of pathways involved in persistent HIV-induced immune dysregulation during suppressive ART.

## Introduction

In the past two decades, the scale-up of antiretroviral therapy (ART) has led to dramatic improvements in life expectancy of people with HIV-1 (PWH) globally. ART effectively and sustainably suppresses viral replication, which allows for immune reconstitution. However, despite sustained ART-mediated viral suppression, 30–60% of PWH have incomplete CD4^+^ T-cell recovery, putting them at a persistently greater risk of AIDS and non-AIDS complications [1–3]. Apart from the recognized risk factors such as older age, male sex, and low pre-ART CD4^+^ cell counts because of advanced HIV disease, the mechanisms underlying incomplete CD4^+^ T-cell recovery remain to be further elucidated [1,2].

Emerging evidence shows that host microRNAs, a group of small, conserved, noncoding RNA molecules of 17–25 nucleotides that modulate gene expression posttranscriptionally by targeting mRNAs, play an important role in HIV pathogenesis and disease progression [4,5]. During HIV infection, microRNAs have been shown to directly target viral transcripts and modulate host pathways involved in the viral life cycle, and hereby influence viral replication and immune responses [6,7]. MicroRNAs have been shown to be differently expressed between PWH and uninfected individuals in body fluids, tissues, and cells [8–11], and have been suggested as potential biomarkers of HIV disease progression [4]. We hypothesized that plasma microRNA profiling could provide novel insights into pathways underlying poor CD4^+^ T-cell recovery during ART-mediated viral suppression.

This study sought to identify host microRNAs in plasma, and their biological pathways, that were associated with poor CD4^+^ T-cell recovery during suppressive ART. To this end, we adopted broad microRNA profiling before and during ART, followed by validation of promising microRNA candidates, within an existing well characterized, multinational cohort of PWH in sub-Saharan Africa [1,12]. Lastly, we assessed to what extent dysregulation of specific microRNAs persisted during suppressive ART, relative to HIV-negative controls.

## Methods

### Study design and participants

This study was nested within the Pan-African Studies to Evaluate Resistance Monitoring (PASER-M) multinational cohort of HIV-1-positive adults (≥18 years) initiating ART, established between 2007 and 2015, as described elsewhere [1,12]. This study included participants from Kenya, Nigeria, South Africa, Uganda, and Zambia who initiated first-line nonnucleoside reverse transcriptase inhibitor-based ART, who had undetectable plasma HIV-RNA (<50 copies/ml) at 12 months after ART initiation, and for whom paired stored plasma samples were available both pre-ART (D0) and at 12 months after ART initiation (M12). Participants with chronic hepatitis B (based on screening for HBsAg seropositivity) or tuberculosis infection (based on local standard-of-care clinical assessment) at D0 were excluded.

First, we randomly selected a small identification cohort (*n* = 24) based on the extent of CD4^+^ T-cell count recovery during the first 12 months of ART (the difference between M12 and D0: ΔCD4): 12 participants from the lowest ΔCD4 tertile (‘poor immune recovery’, PIR) were matched to 12 participants from the highest ΔCD4 tertile (‘good immune recovery’, GIR), based on age, sex, country, pre-ART CD4^+^ T-cell count, and ART regimen using nearest neighbor matching. We performed broad microRNA screening in these 24 individuals at D0 and M12. Subsequently, we selected the candidate microRNAs that had different plasma levels between PIR and GIR groups for confirmation in a validation cohort (*n* = 113), comprised of 61 additional cohort participants with PIR and 52 with GIR. We also tested the selected candidate microRNAs at two time points, D0 and M12. Finally, to investigate to what extent dysregulated microRNAs normalized during suppressive ART, the selected microRNAs were also measured in stored plasma samples from 50 HIV-negative blood bank donors from Nigeria, South Africa, and Uganda [13–15].

### Ethics

The study protocol was approved by the research ethics committees at all study sites. All participants provided written informed consent, including for use of stored samples in future approved research.

### Laboratory procedures

RNA isolation from 200 μl EDTA plasma was performed using the miRCURY RNA Isolation Kit (Exiqon, Vedbaek, Denmark), according to the manufacturer's instructions. Isolated microRNA samples were stored at −80 °C until use. In the identification cohort, cDNA was synthesized using miRCURY LNA Universal RT cDNA synthesis Kit (Exiqon). Initial microRNA detection screening was performed using the 384-well Serum/Plasma Focus microRNA PCR Panel (V4.0) and the ExiLENT SYBR Green Master Mix (Exiqon), measuring 179 human miRNAs. An RNA spike-in kit (Exiqon) was used to monitor the efficiency of RNA isolation, cDNA synthesis and PCR amplification. Spike-in outlier values were calculated using Grubbs’ outlier test and a visual inspection of spike-in line plots was performed. None of the spike-in deviated beyond the 95% confidence interval (CI), and no distinct abnormalities were observed in the spike-in line plots. In the confirmation cohort, cDNA synthesis was done using qScriptTM microRNA cDNA synthesis kit (Quanta Biosciences, USA). Individual microRNA RT-qPCR were performed using LightCycler 480 SYBR Green I Master (Roche Diagnostics, Switzerland). MicroRNA primer sequences were determined using mirBase.org [16] (Table S1). Determination of the threshold cycle (*C*_T_) and the melting curve analysis for the microRNAs were done using Lightcycler 480 software (Roche Diagnostics).

### Data analysis

Differences between groups were tested using the Student's *t* test or Mann–Whitney *U* test, based on the results of the KS-normality. In the identification cohort (*n* = 24), plasma microRNAs were normalized using global mean normalization (CTnorm microRNA of interest, sample A = CT microRNA of interest, sample A – average CT all microRNAs, sample A) and suitable reference genes for the confirmation phase (hsa-miR-30d-5p and has-miR-23–5p) were found by applying the NormFinder and GeNorm algorithms on the plasma microRNA profile panel results. Missing data frequency was determined and microRNAs with less than 60% of samples showing amplification were removed from the analysis (*n* = 1, hsa-miR-208-3p). For all other microRNAs, amplification was at least 60% (median 100%, IQR 96–100%). In the validation cohort, plasma microRNAs were normalized using the ΔCT method (= CT microRNA of interest – CT algorithmic mean of hsa-miR-23-5p and miR-30d-5p). Individual data points were expressed as relative levels using 2^^-ΔCT^. The difference in levels of microRNAs between study groups in the identification cohort was calculated using the comparative CT-method (=2^ ^–(average^^ΔCT^^microRNA^^of^^interest^^PIR-group)^^–^^(average^^ΔCT^^microRNA^^of^^interest^^GIR-group)^) [17] and expressed as the fold change. A negative (positive) fold change signifies a higher (lower) level of the respective microRNA in PIR compared with GIR. Student's *t* test or Mann–Whitney *U* test was used to calculate whether differences were statistically significant. From the microRNAs measured in the identification cohort, we first selected the microRNAs with fold change greater than 2, we then ranked the microRNAs based on *P* value, and, finally, we selected the top 11 microRNAs (top 10 and 1 extra in case one of the microRNAs could not amplify in the individual qPCR) for further analysis. Patient and microRNA patterns and clusters were explored using a heat map (Morpheus, Broad Institute, Boston, USA), principal component analysis, and pairwise correlation.

The 11 selected microRNAs were measured in the validation cohort (*n* = 113), and multivariable logistic regression analysis was used to assess associations between poor CD4^+^ T-cell recovery and plasma microRNA levels (relative levels were log_2_ transformed; log_2_[2^^-ΔCT^]), adjusting for pre-ART CD4^+^ T-cell count, age, sex, HIV subtype, and clustering within countries. Lastly, to assess differences in microRNA levels between PWH and uninfected controls we used the comparative CT-method. MicroRNAs with a fold change greater than 2 and *P* less than 0.05 were considered significantly different between study groups. Statistical analyses were performed with GenEx 6 pro software (MultiD Analyses, Sweden) or Stata 12 (StataCorp, College Station, Texas, USA).

For microRNAs that were differently expressed as identified by the multivariable logistic regression analysis between PIR and GIR study groups in the validation cohort, gene targets were identified using mirTargetLInk 2.0 [18], based on strong evidence, backed up by strong experimental methods like ’reporter gene assay’. Next, for identified genes with strong evidence, we performed a pathway analysis using miRpathDB v2.0 [19] using the Reactome biological pathways database [20]. Pathways considered significantly enriched contain significantly more targets of the respective microRNA than expected by chance, calculated using an over-representation analysis [21]. Finally, we consulted the Gene Expression Omnibus [22], dataset: GSE6740 [23] to explore whether the identified microRNA gene targets were differently expressed in CD4^+^ T cells of people with chronic HIV compared with uninfected controls using GEO2R [22].

## Results

### Study participants’ characteristics

Participants’ characteristics are summarized in Table S2. In the identification cohort, there were no differences for age, sex, country, and ART regimen, whereas (despite matching) pre-ART CD4^+^ T-cell counts were higher among the participants with GIR than with PIR [211 cells/μl (IQR 164–241) vs. 159 cells/μl (IQR 76–202); *P* = 0.035]. In the validation cohort, there were no significant differences between groups. We confirmed that the trajectories of CD4^+^ T-cell recovery remained higher in participants with GIR than PIR beyond the first year (Figure S1). Among the HIV-negative controls, the age distribution was similar, whereas men were over-represented compared with PWH.

### Identification of candidate microRNAs (identification cohort)

**Fig. 1:**
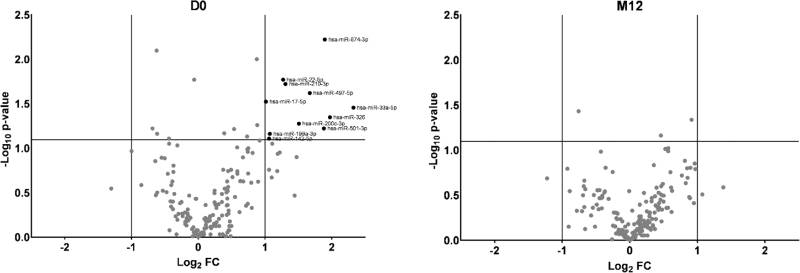
Differentially expressed microRNAs at D0 and M12 between poor immune recovery and good immune recovery groups in the identification cohort.

In the broad microRNA screening at D0 and M12 in the identification cohort, we identified 23 microRNAs with fold change greater than 2. Next, the microRNAs were ranked based on their *P* value and the top 11 was selected for further analysis (Fig. 1 and Table S3). Notably, all selected microRNAs were from D0. A heatmap suggested that relative levels of the selected microRNAs were lower for the PIR group than the GIR group at D0 but seems to be particularly driven by a small subset of participants (Figure S2). In the principal component analysis, moderate clustering was observed among GIR and among PIR, although some spread can be observed for PIR participants (Figure S2). Pairwise correlations for selected microRNAs at D0 were strong (*R* ≥ 0.7) for hsa-miR-142-5p and hsa-miR-199a-3p; hsa-miR-142-5p and hsa-miR-210-3p; hsa-miR-142-5p and hsa-miR-326; hsa-miR199a-3p and hsa-miR-200c-3p; hsa-miR199a-3p and hsa-miR-210-3p; hsa-miR199a-3p and hsa-miR-326; hsa-miR-326 and hsa-miR-33a-5p (Figure S2).

Volcano plot of the log_2_ fold change and −log_10_*P* value. Fold change was calculated between groups using the comparative CT-method {=2 ^^−[(average^^ΔCT^^microRNA^^of^^interest^^in^^PIR-group)^^−^^(average^^ΔCT^^microRNA^^of^^interest^^in^^GIR-group)]^}. Differences between PIR and GIR groups were tested using Student's *t* test or Mann–Whitney, based on the results of the KS-normality test (Supplementary Data). A negative fold change means a higher level of the respective microRNA in the PIR group compared with the GIR group, and vice versa. Black dots present microRNAs selected for further analysis in confirmation cohort. Lines are set at (-)2 FC [= (-)1 log_2_ FC] and *P* value 0.08 (=1.096 – log_10_*P* value), to indicate the threshold for the 11 microRNAs with FC greater than 2 and lowest *P* value. D0, day zero, pre-ART; FC, fold change; GIR, good immune recovery; M12, 12 months after ART start; PIR, poor immune recovery.

### Confirmation of selected microRNAs (validation cohort)

**Fig. 2:**
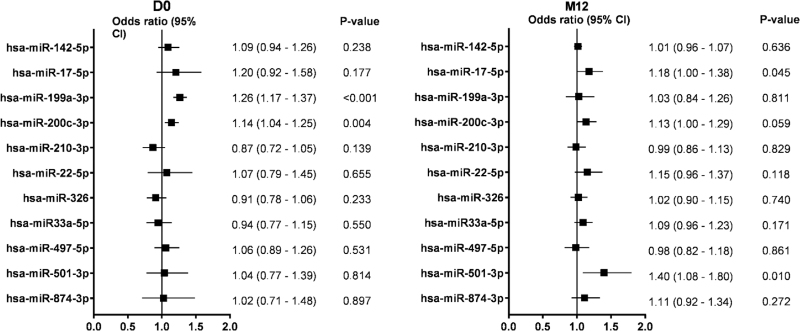
Associations between plasma microRNA levels and poor CD4^+^ T-cell recovery in the validation cohort.

Relative values of the selected microRNAs in the validation cohort are summarized in Figure S3. In the multivariable logistic regression analysis, higher levels of hsa-miR-199a-3p and hsa-miR-200c-3p at D0 were associated with a significantly increased risk of subsequent poor CD4^+^ T-cell recovery during ART [odds ratio (OR) 1.26, 95% CI 1.17–1.37, *P* < 0.001; and OR 1.14, 95% CI 1.04–1.25, *P* = 0.004, respectively; Fig. 2]. For quality control, we conducted a limited assay comparison (focus panel vs. individual RT-qPCRs) by including 12 random identification cohort participants (PIR *n* = 6, GIR *n* = 6) in the RT-qPCR analysis of the validation cohort participants; the direction of the fold changes for hsa-miR-199a-3p and hsa-200c-3p were found to be the same in both assays.

The figure shows the results of the multivariable logistic regression analysis of the association between poor CD4^+^ T-cell recovery and 11 selected microRNAs measured in the confirmation cohort (a) at D0 (pre-ART) and (b) at M12 (month 12). Odds ratio greater than 1 means that for every point increase of the relative microRNA level the odds of having poor CD4^+^ T-cell recovery increases, and vice versa. The model was adjusted for pre-ART CD4^+^ T-cell count, age, sex and HIV subtype, and clustering of observations within country.

In the validation cohort at M12, higher levels of hsa-miR-17-5p and hsa-miR-501-3p were associated with poor CD4^+^ T-cell recovery (OR 1.18, 95% CI 1.00–1.38, *P* = 0.045; and OR 1.40, 95% CI 1.08–1.80, *P* = 0.010, respectively), whereas hsa-miR-199a-3p (OR 1.03, 95% CI 0.84–1.26, *P* = 0.811), hsa-miR-200c-3p (OR 1.13, 95% CI 1.00–1.29, *P* = 0.059) or any of the other microRNAs were not independently associated (Fig. 2).

### MicroRNA plasma levels of HIV-positive individuals compared with HIV-negative controls

Additionally, we investigated differences in plasma microRNA levels between PWH study groups and HIV-negative controls. Plasma levels of hsa-miR-326, hsa-miR-497-5p and hsa-mir-501-3p were significantly lower across all PWH study groups at both time points (PIR and GIR both at D0 and M12) compared with HIV-negative controls. Plasma levels of hsa-miR-199a-3p and miR-200c-3p were higher for all PWH study groups at both time points, except GIR at M12, compared with HIV-negative participants (Table 1 and Figure S3).

**Table 1:** MicroRNA plasma level fold changes between people with HIV and HIV-uninfected controls.

	D0 PIR vs. HIV-uninfected		D0 GIR vs. HIV-uninfected		M12 PIR vs. HIV-uninfected		M12 GIR vs. HIV-uninfected	
	Fold change	*P* value	Fold change	*P* value	Fold change	*P* value	Fold change	*P* value
hsa-miR-142-5p	−1.987	0.002	−1.677	0.017	−1.589	0.037	−1.483	0.098
hsa-miR-17-5p	1.073	0.801	1.642	0.021	−1.289	0.250	1.182	0.511
hsa-miR-199a-3p	−3.597	<0.001	−2.116	0.001	−2.142	<0.001	−1.886	0.008
hsa-miR-200c-3p	−3.645	<0.001	−2.568	<0.001	−2.408	<0.001	−1.827	0.008
hsa-miR-210-3p	1.992	0.019	1.443	0.256	1.579	0.132	1.523	0.077
hsa-miR-22-5p	1.434	0.060	1.492	0.061	1.285	0.227	1.55	0.034
hsa-miR-326	44.674	<0.001	36.752	<0.001	53.082	<0.001	53.857	<0.001
hsa-miR-33a-5p	−1.168	0.273	−1.359	0.068	1.054	0.685	1.416	0.482
hsa-miR-497-5p	2.263	0.003	2.398	0.001	4.246	<0.001	3.914	<0.001
hsa-miR-501-3p	6.185	<0.001	6.196	<0.001	2.704	<0.001	6.064	<0.001
hsa-miR-874-3p	1.181	0.513	1.227	0.438	1.231	0.604	1.328	0.241

Fold change (FC) was calculated between groups using the comparative CT-method {=2 ^ – [(average ΔCT miRNA of interest in PIR-group) – (average ΔCT miRNA of interest in GIR-group)]. Differences between groups were tested using students *t* test or Mann–Whitney}. A negative FC means a higher level of the respective microRNA in the PIR group compared with the GIR group, and vice versa. Bold and shaded FCs and *P* values indicate a significant difference (FC >2 and *P* value <0.05). *C*_T_, cycle threshold; D0, pre-ART; FC, fold change; GIR, good immune recovery; M12, month 12 after ART initiation; PIR, poor immune recovery; PWH, persons with HIV.

### MicroRNA targets and pathway analysis

**Fig. 3:**
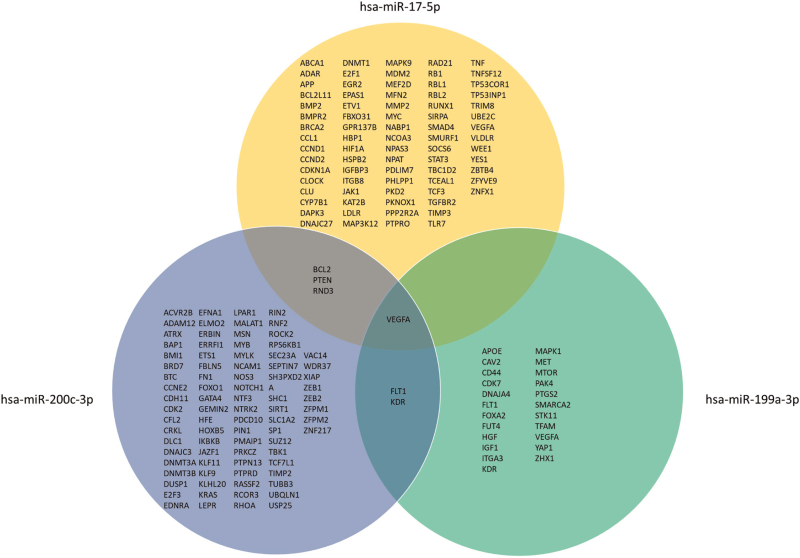
mRNA targets of hsa-miR-17-5p, hsa-miR-199a-3p and hsa-miR-200c-3p.

**Fig. 4:**
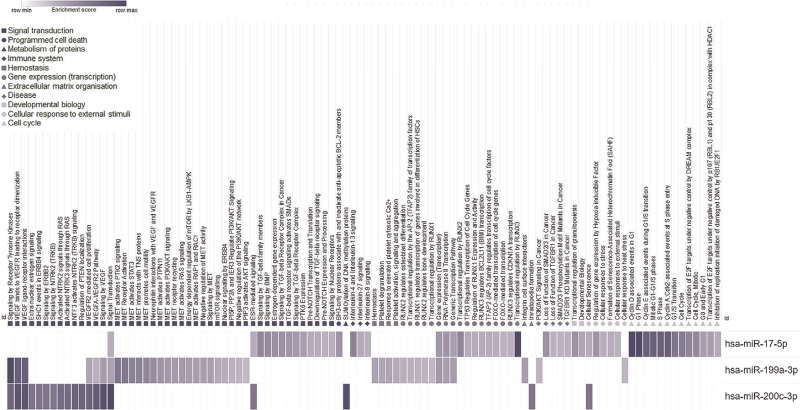
Pathways enriched for gene targets of hsa-miR-17-5p, hsa-miR-199a-3p and hsa-miR-200c-3p.

To explore possible mechanisms influenced by hsa-miR-199a-3p, hsa-200c-3p, hsa-miR-17-5p and hsa-miR-501-3p, we conducted a pathway analysis. We identified mRNA gene targets supported by strong evidence as follows: 23 for hsa-miR-199a-3p, 89 for hsa-miR-200c-3p, 80 for hsa-miR-17-5p and none for hsa-miR-501-3p. Vascular endothelial growth factor A (*VEGFA*) was identified as a target for hsa-miR-17-5p, hsa-miR-199a-3p, and hsa-200c-3p; fms-related receptor tyrosine kinase 1 (*FLT1*) and kinase insert domain receptor (*KDR*) were identified as targets for hsa-miR-199a-3p, and hsa-200c-3p; B-cell lymphoma 2 (*BCL2*), Phosphatase and tensin homolog (*PTEN*) and Rho family GTPase 3 (*RND3*) were identified as targets for both hsa-miR-17-5p and hsa-miR-199a-3p (Fig. 3). Pathway enrichment analysis identified 53 Reactome pathways significantly enriched for targets of hsa-miR-17-5p, 41 pathways for hsa-miR-199a-3p, and 19 pathways for hsa-miR-200c-3p (Tables S4, S5 and S6). The pathways identified were predominantly involved in signal transduction, of which all in tyrosine kinases signaling [6 in signaling by VEGF and 12 in signaling by MET (MNNG-hos transforming gene)], and pathways involved in gene expression, all 17 of which in RNA polymerase II transcription (Fig. 4). Using the GSE6740 dataset [23] available at gene expression omnibus [22] we observed that in CD4^+^ T cells from PWH some of the potential microRNA targets were differently expressed; the miR-17–5p targets *APP*, *CDKN1A*, *EGR2*, *PTPRO*, *TGFBR2*, *MEFD2* were upregulated and *LDLR*, *MDM2*, *IGFBP3* were down regulated; the miR-199a3p targets *CD44*, *CAV2* were upregulated and *FOXA2* was down regulated; the miR-200c-3p targets *FN1*, *KLF9*, *VAC14*, *SLC1A2* were upregulated and *SP1*, *HFE*, *PAIMP1* were downregulated (Figure S4, Table S7, S8 and S9) [23].

Venn diagram for targets of hsa-miR-17-5p, hsa-miR-199a-3p and hsa-miR-200c-3p. Targets were identified using miRTargetLink 2.0. Depicted targets represent targets determined by strong experimental methods. Targets of hsa-miR-17-5p are depicted in the yellow circle, targets of hsa-miR-199a-3p are depicted in the green circle, and targets for hsa-miR-200c-3p are depicted in the blue circle. The overlapping areas depict targets the microRNAs have in common. No targets for miR-501-3p were identified.

Heatmap of pathways significantly enriched for targets of hsa-miR-17-5p, hsa-miR-199a-3p, and hsa-miR-200c-3p. The displayed enrichment scores are computed from the respective *P* values as: enrichment score = −log_10_(*P* value of pathway). Darker colors indicate higher enrichment scores, whereas white indicates no enrichment.

## Discussion

In this study among African PWH who had started ART during severe CD4^+^ T-cell depletion, we identified specific plasma microRNAs that were associated with poor CD4^+^ T-cell recovery despite achieving ART-mediated viral suppression, and explored possible functional biological pathways. Using a robust two-stage approach, we screened 179 microRNAs in an identification cohort of PWH, followed by confirmatory testing of 11 selected candidate microRNAs in a larger validation cohort of PWH. In the validation cohort, we found that increased plasma levels of hsa-miR-199a-3p and hsa-miR-200c-3p measured before ART start, and increased plasma levels of hsa-miR-17-5p and hsa-miR-501-3p measured during suppressive ART, were associated with an increased risk of poor CD4^+^ T-cell recovery.

Our study findings are in line with previous studies that reported hsa-miR-199a-3p to be downregulated in peripheral blood mononuclear cells (PBMCs) of PWH with high CD4^+^ T-cell counts and low viral loads, as compared with those with low CD4^+^ T-cell counts and/or high viral loads [24]. Furthermore, in a previous study in HIV elite controllers, an elevated plasma hsa-miR199a-3p level was predictive of subsequent loss of viral control [25]. In in-vitro studies, miR-199a-3p was upregulated in HIV-1-infected cells compared with cells infected with a Vpr/Vif-deficient HIV-1 strain [26]. The HIV-1 accessory proteins Vpr and Vif are essential in causing cell-cycle arrest in the G2 phase of the cell cycle, which is optimal for the transcription of the viral genome [27–29]. Thus, increased pre-ART levels of hsa-miR-199-3p could be reflective of transcriptional activity in participants who experience subsequent poor immune recovery during ART.

Hsa-miR-200c-3p has previously been shown to be higher in PBMCs from HIV progressors than HIV elite controllers and HIV-uninfected controls [30]. Our pathway analysis identified several targets (e.g. BCL2, ZEB1 and ZEB2) that play a role in apoptosis, and several studies have linked hsa-miR-200c-3p to apoptosis through its targets [31,32]. Our findings could, therefore, be suggestive of increased apoptotic activity in PWH who experience poor CD4^+^ T-cell recovery.

Regarding hsa-miR-501-3p, our findings concur with a recent study that found that hsa-miR-501-3p was downregulated in CD4^+^ T cells from natural HIV controllers (i.e. those maintaining a viral load <2000 copies/ml without ART) compared with typical HIV progressors [33]. Although little else is known about hsa-miR-501-3p in relation to HIV, it has been suggested that it may promote hepatitis B virus replication [34] and infectivity, and viral assembly in hepatitis C virus *in vitro*[35]. Although the underlying mechanisms of the relation between hsa-miR-501-3p and CD4^+^ T-cell recovery remains to be elucidated, these findings suggest that hsa-miR-501-3p could be reflective of ongoing viral replication.

We found that hasa-miR-17-5p was associated with poor CD4^+^ T-cell recovery during ART. Hsa-miR-17-5p has previously been associated with HIV replication; in-vitro assays showed that increasing the levels of miR-17-5p caused reduced HIV replication, whereas inhibition of miR-17-5p increased replication [7].

Our findings are incongruent with a recent study in Spain that found lower levels of miR-106a and miR-140 and higher levels of miR-192 in individuals with poor CD4^+^ T-cell recovery compared with those with good CD4^+^ T-cell recovery[36]. The discrepant findings could be a result of the different compartments studied: the study in Spain focused specifically on exosome-derived microRNAs, whereas our study isolated total microRNA, which includes microRNAs from extracellular vesicles as well as vesicle-free microRNAs (e.g. RNA-protein complexes). The distribution and composition of microRNAs may differ between different vesicles and vesicle-free fractions [37]. Additionally, differences between the study populations, such as host genetic make-up, HIV disease status, pathogen exposure histories and other environmental exposures could have influenced circulating microRNAs [38,39].

In our study, we observed that, in comparison with HIV-negative controls, dysregulation of several microRNAs persisted despite successful suppressive ART supporting a potential regulatory role of microRNAs in persistent immune dysregulation in PWH. Compared with HIV-uninfected controls, PWH had lower levels of hsa-miR-326, hsa-miR-497-5p, and hsa-miR-501-3p before and during ART; and they had higher hsa-miR-199a-3p and hsa-miR-200c-3p levels before ART, and, in participants with poor CD4^+^ T-cell recovery, also during ART. Notably, we found that PWH had 37–54-fold lower hsa-miR-326 levels compared with HIV-negative controls, which could reflect the severe depletion of CD4^+^ T cells in our study population prior to starting ART, including the loss of Th17 cell populations [40], of which hsa-miR-326 is a promotor [41]. Additionally, hsa-miR-326 is a potent HIV replication repressor [42], suggesting that the markedly reduced levels of hsa-miR-326 in PWH in this study could signal loss of control of HIV replication by this microRNA.

The pathway analysis suggested that targets of hsa-miR-17-5p, hsa-miR-199a-3p, and hsa-miR-200c-3p are predominantly involved in signal transduction and gene expression (transcription). Specifically, mRNAs encoding for proteins involved in RNA polymerase II are targeted by hsa-miR-17-5p, hsa-miR-199a-3p, and hsa-miR-200c-3p. RNA polymerase II plays a crucial role in HIV transcription [43]. Hence, our findings could reflect ongoing transcriptional activity of HIV-infected cells. Additionally, we found that hsa-miR-17-5p, hsa-miR-199a-3p, and hsa-miR-200c-3p target mRNAs (*VEGFA*, *FLT1*, *KDR*) involved in the VEGF signaling pathway, which –amongst others – plays a role in angiogenesis, vascular permeability, cell proliferation, and cell migration. The HIV-1 tat (Trans-Activator of Transcription) protein, essential in HIV replication, has been shown to target the VEGF receptor KDR [44], and act in synergy with VEGFA [45]. It has been suggested that HIV-1 tat induces nonclassical T-cell activation through VEGF signaling, which promotes HIV-infection susceptibility and viral replication *in vitro*[35]. However, a relationship, if any, between HIV-1 tat, VEGF-signaling and hsa-miR-17-5p, hsa-miR-199a-3p and hsa-miR-200c-3p, in the context of CD4^+^ T-cell recovery, remains to be elucidated. Furthermore, hsa-miR-199a-3p targets *MET*, *ITGA3* and *HGF*, which are involved in the MET signaling pathway. The MET signaling pathway has been predominantly described in cancer, and a key role is the promotion of cell survival. Interestingly, HIV has been shown to increase the expression of *ITGA3* in macrophages to promote viral replication [46]. Whether the increased levels of hsa-miR-199a-3p in plasma of participants with poor CD4^+^ T-cell recovery reflects the interaction with ITGA3 and its role in viral replication, requires further investigation.

The analysis of the gene expression dataset: GSE6740, indicated that some of the miR-17-5p, miR-199a-3p and miR-200c-3p gene targets were differently expressed in CD4^+^ T cells of PWH compared with uninfected controls. *APP*, *CDKN1A*, *EGR2*, *PTPRO*, *TGFBR2*, *MEFD2* were upregulated and *LDLR*, *MDM2*, *IGFBP3* were down regulated; *CD44*, *CAV2* were upregulated and *FOXA2* was down regulated; *FN1*, *KLF9*, *VAC14*, *SLC1A2* were upregulated and *SP1*, *HFE*, *PAIMP1* were downregulated. Some of these genes have been suggested to play a distinct role in HIV infection. SP1 is known to be a key regulator of HIV transcription in HIV-infected cells [47]. In addition, LDLR2 is crucial in cholesterol metabolism, which is altered by HIV to create favorable conditions for HIV replication [48]. Finally, MDM2 plays a key role in HIV-infected CD4^+^ T-cell survival, through p53 degradation [49]. Modulation of the expression of these genes in CD4^+^ T cells may, therefore, regulate HIV-replication. Whether these genes are indeed degraded through microRNA–mRNA interaction remains to be elucidated.

Finally, the pathway analysis did not identify gene targets with strong evidence for miR-501-3p. Some gene targets with weak evidence can be found in the miRtargetLink 2.0 database. Of particular interest is beta-2-microglobulin (B2M), of which plasma and serum levels have previously been associated with HIV and disease progression [50]. In addition, β2 microglobulin protein is a component of the major histocompatibility complex class I (MHC-1) molecule, which plays an important role in antigen presentation and is down regulated by the HIV Nef accessory protein [51].

There are several study limitations. First, as there were no PBMCs available for this cohort, we were only able to measure microRNAs in the plasma compartment. Circulating microRNAs are derived from both circulating cells as well as tissues [52]. Additionally, not all microRNAs are secreted in the circulation in equal amount and manner, for example, released because of0 cytolysis or tissue injury, in apoptotic bodies or actively secreted from cells in exosomes and other microvesicles, or as RNA-protein complexes [53]. The microRNA tissue atlas (https://ccb-web.cs.uni-saarland.de/tissueatlas2) indeed shows that a wide variety of cells and tissues express the microRNAs found in this study [54,55]. Within the PBMC compartment, hsa-miR-17-5p, hsa-miR-200c-3p and hsa-miR-501-3p are expressed in particular by CD14^+^ cells, whereas hsa-miR-199a-3p is expressed in particular by CD15^+^ cells [55]. Furthermore, the maturation and polarization of monocytes may also alter the expression profile of microRNAs [56]. It has been well described that microRNA expression in PBMCs also changes during HIV infection. In CD4^+^ T cells of PWH, compared with uninfected controls, no differences in expression of hsa-miR-17-5p, hsa-miR-199a-3p, hsa-miR-200c-3p or hsa-miR-501-3p have been observed [57]. However, given that in PWH, HIV-1 infects only a small fraction of the circulating CD4^+^ T cells, it is possible that the observed levels of microRNAs in plasma do not find their origin in CD4^+^ T cells and instead are likely to be the result of indirect bystander effects such as systemic changes in immune activation and inflammation caused by the HIV infection. For example, in a previous study miR-200c-3p and miR-17-5p was upregulated in PBMCs from viremic progressors and ART-treated PWH compared with Elite Controllers and uninfected controls [30], and hsa-miR-199a-3p was downregulated in PBMCs from PWH with high CD4^+^ T cells and a low viral load compared with uninfected controls, but not in PBMCs of PWH who had a low CD4^+^ T-cell count or high viral load [42]. In addition, a study which investigated the expression level of microRNAs in adipose tissue showed that miR-199a-3p was higher in tissue of PWH compared with uninfected controls [9].

Second, although the pathway analysis indicated possible targets for the identified microRNAs, it is unknown which cells are targeted by the circulating microRNAs. It is thought that microRNAs play a role in cell–cell communication; however, the uptake mechanisms of extracellular microRNAs are not well understood. The uptake mechanism of vesicle-free microRNA (e.g. RNA-protein complexes) has been proposed to take place through specific receptors on the cell surface, whereas microRNAs in vesicles enter cells through phagocytosis, endocytosis or fusion with the plasma membrane [58].

Third, despite adjustment for potential confounders (e.g. age, sex, country, baseline CD4^+^ T-cell count and excluding participants with chronic hepatitis B or tuberculosis), we cannot rule out residual confounding effects on microRNA levels. Similarly, limited available information on the HIV-negative blood donors meant that possible unknown confounding could not be ruled out [14,15]. Lastly, an inconsistent finding in our dataset was that pre-ART hsa-miR-199a-3p and hsa-miR-200c-3p levels in participants with PIR were found to be increased in the validation cohort (in multivariable analysis), whereas they were decreased in the identification cohort; given that identification (Focus panel) and validation (RT-qPCR) assays showed similar results in the dual-tested samples, the observed inconsistency is most likely because of chance because of the small size of the identification cohort, although residual confounding factors cannot be ruled out.

In conclusion, this analysis suggested that various plasma microRNAs are associated with poor CD4^+^ T-cell recovery during ART-mediated viral suppression. Pathway analysis of hsa-miR-17-5p, hsa-miR-199a-3p and hsa-miR-200c-3p suggested a possible role for signal transduction pathways, specifically VEGF and MET signaling, as well as pathways involved in gene expression, specifically RNA polymerase II. These findings add to the understanding of microRNAs during HIV infection and suggest possible biological pathways involved in persistent HIV-induced immune dysregulation during treated HIV infection.

## Acknowledgements

The authors thank the study participants, the staff at the collaborating clinical sites and reference laboratories.

PASER-M collaborators: Lusaka Trust Hospital (M. Siwale), Coptic Hospital (M. Labib), KARA Clinic and Laboratory (J. Menke), Lusaka, Zambia; Muelmed Hospital, Pretoria, South Africa (M. E. Botes [deceased], M. de Jager); Themba Lethu Clinic, Clinical HIV Research Unit, (P. Ive, and I. Sanne) and Department of Molecular Medicine and Haematology (E. Letsoalo, W.S. Stevens, K. Steegen), University of the Witwatersrand, Johannesburg, South Africa; Acts Clinic, White River, South Africa (M. Hardman); Newlands Clinic, Harare, Zimbabwe (M. Wellington, R. Luthy); Coast Province General Hospital, International Centre for Reproductive Health Kenya, Mombasa, Kenya (K. Mandaliya); Mater Misericordiae Hospital, Nairobi, Kenya (M. Dolan); Joint Clinical Research Centre, Fort Portal, Mbale and Kampala, Uganda (C. Kityo, S. Balinda, W. Namala, H. Namata, F. Senono, R. Nakanjako, M. Mutebi, I. Nankya, P. Mugyenyi); Lagos University Teaching Hospital, Lagos, Nigeria (A. Osibogun, A.S. Akanmu, T. Adeyemo, T. Rodoye, H. Adelabu); Amsterdam Institute for Global Health and Development, Kampala, Uganda (C. Nalubwama, H. Kakooza, M. Nakitto, M. O’Mello); Department of Global Health, Amsterdam UMC of the University of Amsterdam, Amsterdam Institute for Global Health and Development, Amsterdam, the Netherlands (R.L. Hamers, K.C.E. Sigaloff, T.S. Boender, S. Inzaule, P. Ondoa, C. Manting-de Vries, N. Pakker, F.W. Wit, J.M. Lange [deceased], T.F. Rinke de Wit).

Funding: this work was part of the M-PACT (Markers of Persistent Immune Activation during Antiretroviral Therapy in Africa) study, supported by a Veni postdoc fellowship to R.L.H. through the Dutch Research Council (NWO) Talent Programme (91615036), and Gilead Sciences Netherlands through an unrestricted scientific grant.

The PanAfrican Studies to Evaluate Resistance (PASER) is an initiative of the Amsterdam Institute for Global Health and Development, with major support provided by the Ministry of Foreign Affairs of The Netherlands through a partnership with Stichting Aids Fonds (12454), and with additional support from De Grote Onderneming, The Embassy of the Kingdom of the Netherlands, Heineken Africa Foundation, Jura Foundation, and the Netherlands Organization for Scientific Research through the Netherlands-African Partnership for Capacity Development Clinical Interventions against Poverty-Related Diseases (W07.10.101 and W07.10.106).

Authors’ contributions: R.L.H. is the M-PACT principal investigator. T.F.R.W. is the PASER principal investigator. C.M.K., M.S., S.A., K.M., M.d.J., T.F.R.W. and R.L.H. established the cohort and supervised data collection. S.K., N.A.K. and R.L.H. conceived the immunology study. S.K. performed the laboratory testing, with help from A.v.N., and supervised by N.A.K. S.K. performed the statistical analyses, with advice from N.A.K., F.W.W. and R.L.H. S.K., N.A.K. and R.L.H. drafted the manuscript. All authors provided valuable input to interpretation of the data and critically reviewed the article for important intellectual content. All authors reviewed and approved the final version of the manuscript.

### Conflicts of interest

P.R. through his institution has received independent scientific grant support from Gilead Sciences, Janssen Pharmaceuticals Inc, Merck & Co, and ViiV Healthcare; he has served on scientific advisory boards for Gilead Sciences, ViiV Healthcare, and Merck & Co, for which his institution has received remuneration, all unrelated to the current manuscript. T.R. received travel support from Merck and payment for lectures from Merck and Abbott, all unrelated to the current manuscript. R.L.H. through his institution has received independent scientific grant support from Gilead Sciences Netherlands to support the microRNA laboratory testing in this study. All other authors declare that they have no conflicts of interests.

## Supplementary Material
