# Supplementary material for: Specific plasma microRNAs are associated with CD4+ T-cell recovery during suppressive antiretroviral therapy for HIV-1
Source: AIDS. 2024 Feb 1;38(6):791–801. doi: 10.1097/QAD.0000000000003853 (PMC10994156; doi:10.1097/QAD.0000000000003853)
Supplement: Supplementary file 1 [file aids-38-791-s001.docx]

**Supplementary material**

**Specific plasma microRNAs are associated with CD4+ T-cell recovery during suppressive antiretroviral therapy for HIV-1**

**Table of contents**

| **Supplementary material** | **Page** |
| --- | --- |
| Figure S1. Median CD4+ T-cell count trajectories in participants with good versus poor CD4+ T-cell recovery in the validation cohort | 3 |
| Figure S2. Participant and microRNA clusters in the identification cohort for 11 selected microRNAs | 4 |
| Figure S3. Relative microRNA levels in the validation cohort | 6 |
| Figure S4. Expression of target genes in CD4+ T-cells | 8 |
| Table S1. MicroRNA primers | 9 |
| Table S2. Participant characteristics | 10 |
| Table S3. Mean and standard deviation for 179 microRNAs and group comparison in the identification cohort | 11 |
| Table S4. Pathways and targets of hsa-miR-17-5p | 16 |
| Table S5. Pathways and targets of hsa-miR-199a-3p | 19 |
| Table S6. Pathways and targets of hsa-miR-200c-3p | 21 |
| Table S7: Expression of target genes from miR-17-5p in CD4+ T-cells | 22 |
| Table S8: Expression of target genes from miR-199a-3p in CD4+ T-cells | 25 |
| Table S9: Expression of target genes from miR-200c-3p in CD4+ T-cells | 27 |

**Figure S1. Median CD4+ T-cell count trajectories in participants with good versus poor CD4+ T-cell recovery in the validation cohort**

Participants in the lowest tertile of CD4+ T-cell gain (the difference between M12 and D0) were classified as “poor immune recovery” (PIR) and those in the highest tertile as “good immune recovery” (GIR). Dashed lines indicate IQR. Analysis time is expressed in years after start ART. Participants per group were: PIR n= 61, GIR n=52.

Abbreviations: GIR=good immune recovery; PIR = Poor immune recovery.


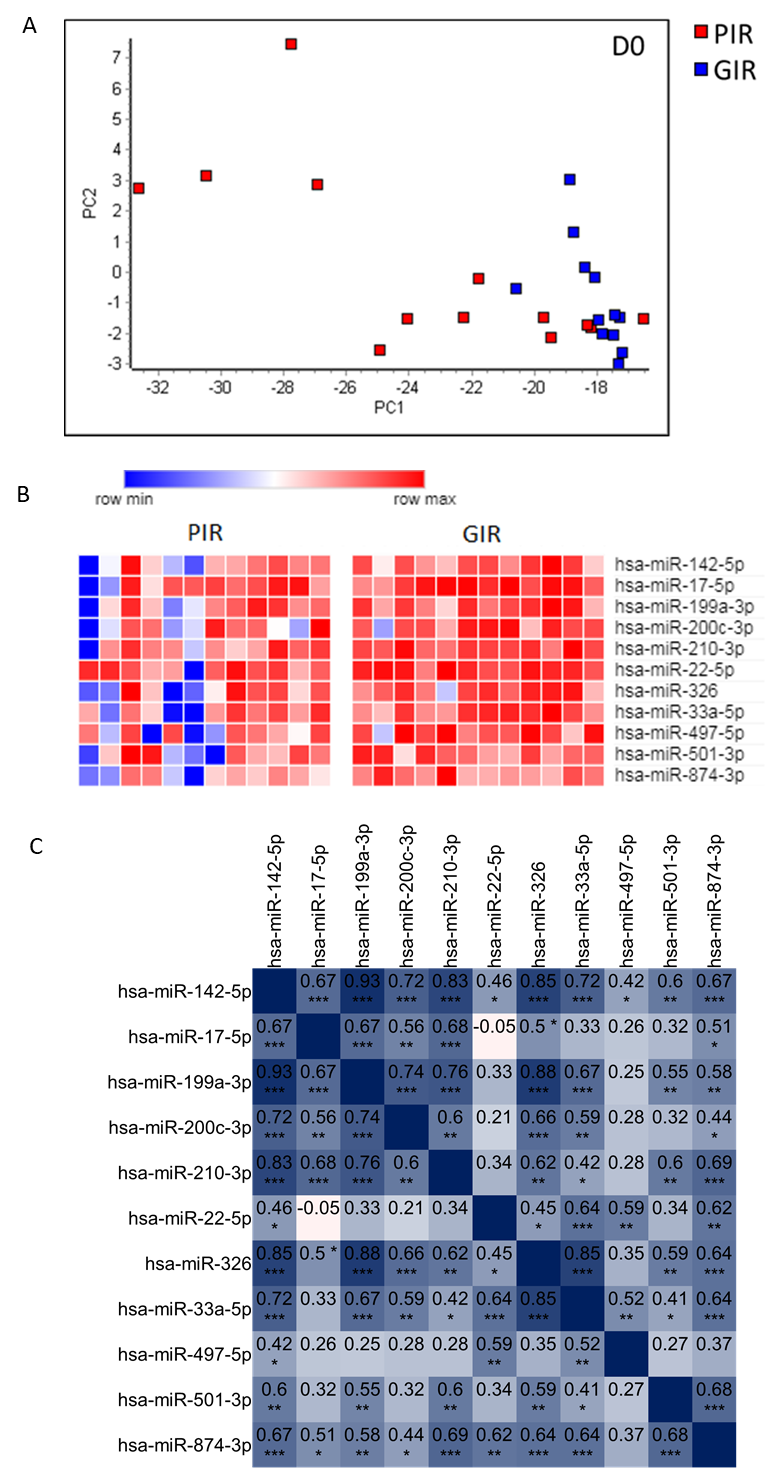


**Figure S2. Participant and microRNA clusters in the identification cohort for 11 selected microRNAs at D0**

A. Principal component analysis of the 11 selected pre-ART microRNA levels (CTnorm values = CT microRNA of interest, sample A – average CT all microRNAs, sample A). Red dots represent participants with poor immune recovery (PIR) and blue dots represent participants with good immune recovery (GIR).

B. Heatmap of 11 selected pre-ART microRNAs levels. Red indicates higher relative microRNA levels, blue indicates lower relative microRNA levels. Colors are based on the values of the maximum and minimum value per rows.

C. Correlation matrix of 11 selected pre-ART microRNAs. We performed pairwise correlation of microRNAs (of CTnorm values). Values are expressed as correlation coefficients. Darker colors indicate stronger correlation, and lighter colors indicate weaker correlation. P-values are indicated by asterisk: >0.05 (*), >0.01 (**), >0.001 (***).

Abbreviations: PIR=Poor Immune Recovery; GIR=Good Immune Recovery.

**Figure S3. Relative microRNA levels in the validation cohort**

Relative microRNA levels in the validation cohort. Dots represent individual data points of relative microRNA levels. Red bars and whiskers represent the median and IQR of relative microRNA levels per study group.

Abbreviations: D0=day zero, pre-ART; GIR=good immune recovery; M12=12 months on ART; PIR=poor immune recovery


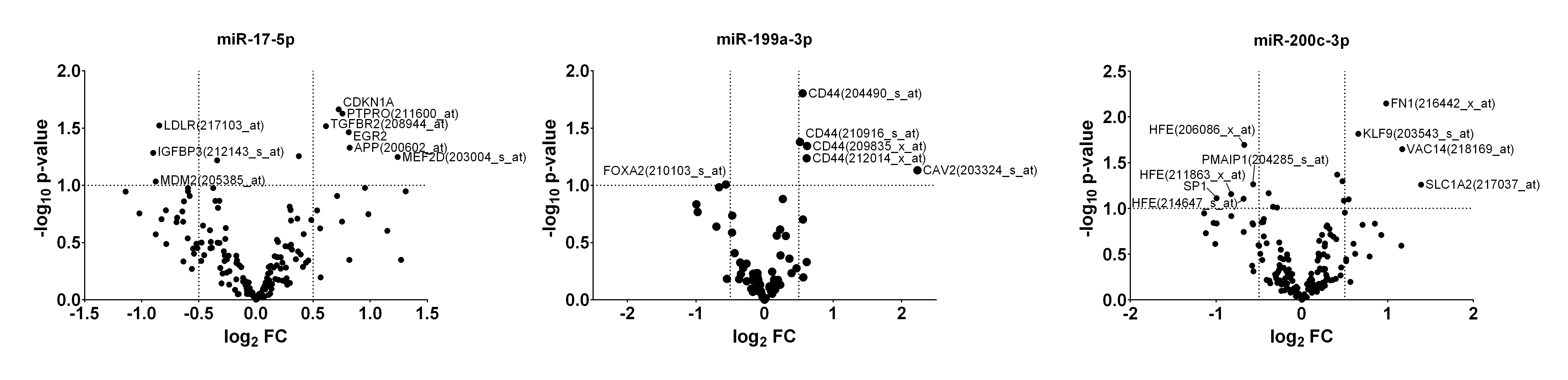


**Figure S4. Expression of target genes in CD4+ T-cells.**

Data represent the differential gene expression of targets of miR-17-5p, miR-199a-3p and miR-200c-3p in CD4+ T-cells of people living with chronic HIV compared with uninfected controls. Data were obtained through Gene Expression Omnibus[22]; dataset GSE6740[23]. A fold change (FC) of >0 indicates upregulation of the genes in CD4+ T-cells from people living with chronic HIV compared with uninfected controls and vice versa.

**Table S1. MicroRNA primers**

| **MicroRNA** | **Sequence** |
| --- | --- |
| hsa-miR-142-5p | CATAAAGTAGAAAGCACTACT |
| hsa-miR-17-5p | CAAAGTGCTTACAGTGCAGGTAG |
| hsa-miR-199a-3p | ACAGTAGTCTGCACATTGGTTA |
| hsa-miR-200c-3p | TAATACTGCCGGGTAATGATGGA |
| hsa-miR-210-3p | CTGTGCGTGTGACAGCGGCTGA |
| hsa-miR-22-5p | AGTTCTTCAGTGGCAAGCTTTA |
| hsa-miR-326 | CCTCTGGGCCCTTCCTCCAG |
| hsa-miR-33a-5p | GTGCATTGTAGTTGCATTGCA |
| hsa-miR-497-5p | CAGCAGCACACTGTGGTTTGT |
| hsa-miR-501-3p | AATGCACCCGGGCAAGGATTCT |
| hsa-miR-874-3p | CTGCCCTGGCCCGAGGGACCGA |
| *hsa-miR-23a-3p* | *ATCACATTGCCAGGGATTTCC* |
| *hsa-miR-30d-5p* | *TGTAAACATCCCCGACTGGAAG* |

Primer sequences were determined using Mirbase.org. MicroRNAs in italic indicate reference microRNAs

**Table S2 Participant characteristics**

|  | **Identification cohort** | |  |  | **Validation cohort** | |  |  |  |
| --- | --- | --- | --- | --- | --- | --- | --- | --- | --- |
|  | **PIR** | **GIR** | **p-value** |  | **PIR** | **GIR** | **HIV-negative** | **PIR vs GIR** | **PLWH vs HIV-negative** |
|  | **(n=12)** | **(n=12)** |  |  | **(n = 61)** | **(n = 52)** | **(n= 50)** | **p-value** | **p-value** |
| **Age** | 39 (31-44) | 40 (27-46) | 0.7944 |  | 36 (34-42) | 36 (30-42) | 36 (30-42) | 0.636 | 0.36 |
| **Sex** |  |  | 1.00 |  |  |  |  | 0.979 | 0.034 |
| Female | 10 (80.0%) | 10 (80.0%) |  |  | 33 (54.1%) | 28 (53.8%) | 18 (36.0%) |  |  |
| Male | 2 (20.0%) | 2 (20.0%) |  |  | 28 (45.9%) | 24 (46.2%) | 32 (64.0%) |  |  |
| **Country** |  |  | 1.00 |  |  |  |  | 0.848 | <0.001 |
| Kenya | 1 (8.3%) | 1 (8.3%) |  |  | 16 (26.2%) | 13 (25.0%) | - |  |  |
| Nigeria | 3 (25.0%) | 3 (25.0%) |  |  | 10 (16.4%) | 5 (9.6%) | 15 (30.0%) |  |  |
| South Africa | 1 (8.3%) | 1 (8.3%) |  |  | 10 (16.4%) | 10 (19.2%) | 15 (30.0%) |  |  |
| Uganda | 7 (58.3%) | 7 (58.3%) |  |  | 20 (32.8%) | 20 (38.5%) | 20 (40.0%) |  |  |
| Zambia | - | - |  |  | 5 (8.2%) | 4 (7.7%) | - |  |  |
| **Pre-ART (D0) CD4+ T cell count cells/µL** | 159 (76-202) | 211 (164-241) | 0.0349 |  | 163 (117-218) | 164 (127-205) | - | 0.685 | - |
| **Month 12 (M12) CD4+ T cell count cells/µL** | 167 (140-189) | 582 (553-668) | <0.0001 |  | 208 (164-268) | 445 (407-502) | - | <0.001 | - |
| **CD4+ T cell count gain cells/µL (D0-M12)** | -3 (-60-59) | 381 (320-481) | <0.0001 |  | 70 (16-89) | 271 (241-336) | - | <0.001 | - |
| **ART regimen** |  |  | 1.00 |  |  |  |  | 0.213 |  |
| **NNRTI** |  |  |  |  |  |  |  |  |  |
| Efavirenz | 4 (33.3%) | 4 (33.3%) |  |  | 29 (47.5%) | 25 (48.1%) |  |  |  |
| Nevirapine | 8 (66.7%) | 8 (66.7%) |  |  | 32 (52.6% | 27 (51.9%) |  |  |  |
| **NRTI** |  |  |  |  |  |  |  |  |  |
| Tenofovir | 6 (50.0%) | 6 (50.0%) |  |  | 20 (32.8%) | 25 (48.1%) |  |  |  |
| Stavudine | 0 (0.0%) | 0 (0.0%) |  |  | 6 (9.8%) | 8 (15.4%) |  |  |  |
| Zidovudine | 6 (50.0%) | 6 (50.0%) |  |  | 34 (55.7%) | 18 (34.6%) |  |  |  |
| Abacavir | 0 (0.0%) | 0 (0.0%) |  |  | 1 (1.6%) | 1 (1.9%) |  |  |  |

Values are presented as median (IQR) or n (%). Differences between study groups were tested using Student’s t-test or Mann-Whitney U test, whichever was appropriate.

Abbreviations: ART= antiretroviral therapy; D0=day zero, pre-ART; GIR=good immune recovery; M12=12 months on ART; NNRTI=non-nucleoside reverse transcription inhibitor; NRTI= non-nucleos(t)ide reverse transcription inhibitor; PIR=poor immune recovery; PLWH=people living with HIV

**Table S3. Mean and standard deviation for 179 microRNAs and group comparison in the identification cohort**

|  | **Mean and standard deviation** | | | | **PIR D0 vs GIR D0** | | **PIR M12 vs GIR M12** | |
| --- | --- | --- | --- | --- | --- | --- | --- | --- |
| **MicroRNA** | **PIR D0** | **PIR M12** | **GIR D0** | **GIR M12** | **Fold change** | **p-value** | **Fold change** | **p-value** |
| hsa-let-7a-5p | 1.97±1.23 | 1.14±0.68 | 1.75±0.44 | 1.34±0.73 | 1.165 | 0.565 | -1.149 | 0.496 |
| hsa-let-7b-3p | 6.36±0.66 | 6.12±0.94 | 6.45±1.18 | 6.39±1.19 | -1.067 | 0.813 | -1.2 | 0.977* |
| hsa-let-7b-5p | 1.63±0.72 | 2.32±0.81 | 2±0.61 | 2.39±1.32 | -1.294 | 0.185 | -1.048 | 0.707* |
| hsa-let-7c-5p | 5.87±0.94 | 5.22±0.98 | 5.47±0.67 | 5.11±0.69 | 1.315 | 0.285* | 1.08 | 0.75 |
| hsa-let-7d-3p | 1.5±0.91 | 1.73±0.73 | 1.41±0.64 | 1.74±0.65 | 1.062 | 0.931* | -1.006 | 0.977 |
| hsa-let-7d-5p | 4.96±1.81 | 4.63±1.25 | 4.64±1.38 | 4.2±1.15 | 1.25 | 0.931* | 1.349 | 0.387 |
| hsa-let-7e-5p | 7.29±1.15 | 6.17±1.27 | 6.7±1.67 | 6.58±1.32 | 1.502 | 0.194* | -1.333 | 0.442 |
| hsa-let-7f-5p | 3.66±0.99 | 2.9±1.2 | 3.82±1.78 | 2.89±0.68 | -1.117 | 0.544* | 1.01 | 0.971 |
| hsa-let-7g-5p | 1.76±0.51 | 1.35±0.59 | 1.37±0.49 | 1.21±0.68 | 1.31 | 0.068 | 1.102 | 0.596 |
| hsa-let-7i-5p | 2.07±1.15 | 1.56±0.46 | 1.64±0.29 | 1.73±0.5 | 1.35 | 0.237* | -1.125 | 0.795* |
| hsa-miR-1 | 7.03±2.68 | 7.79±2.43 | 7.09±2.22 | 7.32±2.1 | -1.041 | 0.954 | 1.384 | 0.618 |
| hsa-miR-100-5p | 8.51±2.33 | 6.53±1.6 | 7.8±2.18 | 7.22±1.7 | 1.642 | 0.445 | -1.615 | 0.317 |
| hsa-miR-101-3p | -0.76±0.65 | -0.96±0.33 | -0.86±0.29 | -0.78±0.54 | 1.076 | 0.61 | -1.133 | 0.507* |
| hsa-miR-103a-3p | 1.12±0.96 | 0.83±1.11 | 0.68±1.33 | 0.45±1.11 | 1.352 | 0.369 | 1.302 | 0.411 |
| hsa-miR-106a-5p | -0.52±0.73 | 0.07±1.03 | -0.69±0.82 | -0.53±1.08 | 1.130 | 0.583 | 1.517 | 0.176 |
| hsa-miR-106b-3p | 7.02±2.4 | 7.2±2.14 | 7.05±2.21 | 7.98±3.01 | -1.02 | 0.840* | -1.721 | 0.471 |
| hsa-miR-106b-5p | 1.9±0.72 | 1.88±0.51 | 2.1±0.81 | 1.74±0.61 | -1.148 | 0.529 | 1.103 | 0.545 |
| hsa-miR-107 | 3.23±1.14 | 2.51±1.18 | 2.48±1.04 | 2.21±0.88 | 1.673 | 0.11 | 1.228 | 0.491 |
| hsa-miR-10b-5p | 6.21±1.91 | 5.54±1.7 | 6.16±1.19 | 5.06±1.38 | 1.034 | 0.942 | 1.392 | 0.457 |
| hsa-miR-122-5p | 3.69±1.62 | 3.73±2.3 | 3.42±1.95 | 3.76±1.81 | 1.208 | 0.470* | -1.021 | 0.971 |
| hsa-miR-125a-5p | 4.92±1.52 | 4.39±1 | 4.11±0.77 | 3.97±0.95 | 1.758 | 0.113 | 1.344 | 0.297 |
| hsa-miR-125b-5p | 4.97±0.89 | 4.84±0.86 | 4.91±0.96 | 4.63±1.08 | 1.037 | 0.885* | 1.155 | 0.608 |
| hsa-miR-1260a | 4.82±1.36 | 5.68±1.12 | 5.24±0.96 | 5.53±1.17 | -1.333 | 0.396 | 1.113 | 0.744 |
| hsa-miR-126-3p | -1.64±0.49 | -1.17±0.76 | -1.33±0.38 | -1.25±0.69 | -1.243 | 0.093 | 1.056 | 0.794 |
| hsa-miR-126-5p | 0.97±0.59 | 1.45±1.01 | 1.13±0.49 | 1.52±0.96 | -1.115 | 0.489 | -1.045 | 0.875 |
| hsa-miR-127-3p | 7.16±2.85 | 8.74±3.18 | 8.01±2.71 | 7.36±2.64 | -1.806 | 0.260* | 2.609 | 0.259 |
| hsa-miR-128-3p | 5.41±1.26 | 5.37±0.85 | 4.68±0.49 | 4.8±0.75 | 1.661 | 0.074 | 1.484 | 0.095 |
| hsa-miR-130a-3p | 3.22±1.53 | 3.39±1.45 | 2.47±1.17 | 2.94±0.81 | 1.678 | 0.193 | 1.369 | 0.355 |
| hsa-miR-130b-3p | 5.74±1.17 | 6.3±1.4 | 5.62±0.95 | 6±0.53 | 1.088 | 0.783 | 1.226 | 0.503 |
| hsa-miR-132-3p | 4.92±0.74 | 5.41±0.67 | 5.07±0.79 | 5.49±1.11 | -1.106 | 0.645 | -1.056 | 0.836 |
| hsa-miR-133a-3p | 6.74±2.27 | 7.26±2.25 | 5.67±2.01 | 6.36±1.91 | 2.086 | 0.175* | 1.861 | 0.157* |
| hsa-miR-133b | 5.67±2.31 | 5.72±1.6 | 4.48±1.01 | 5.22±1.04 | 2.282 | 0.116 | 1.414 | 0.373 |
| hsa-miR-136-3p | 6.69±1.56 | 6.81±1.71 | 6.42±1.67 | 6.62±1.52 | 1.21 | 0.681 | 1.135 | 0.784 |
| hsa-miR-136-5p | 6.4±2.4 | 6.41±2.47 | 5.66±2.01 | 6.33±2.1 | 1.671 | 0.422 | 1.058 | 0.931 |
| hsa-miR-139-5p | 5.09±0.95 | 4.74±0.98 | 4.8±0.78 | 4.72±1.33 | 1.218 | 0.433 | 1.016 | 0.961 |
| hsa-miR-140-3p | 0.66±0.49 | 1.08±0.87 | 0.78±0.6 | 1.1±0.73 | -1.084 | 0.607 | -1.012 | 0.958 |
| hsa-miR-140-5p | 4.86±0.85 | 5.23±1.59 | 4.77±0.94 | 5.27±1.39 | 1.067 | 0.800 | -1.031 | 0.795* |
| hsa-miR-141-3p | 8.08±1.86 | 7.71±2.4 | 7.28±1.36 | 8.42±2.36 | 1.734 | 0.245 | -1.631 | 0.476 |
| hsa-miR-142-3p | 0.38±0.9 | -0.86±1.04 | -0.11±0.82 | -0.48±0.63 | 1.406 | 0.175 | -1.298 | 0.295 |
| **hsa-miR-142-5p** | 2.49±1.56 | 2.13±1.16 | 1.43±0.76 | 2.29±2 | **2.087** | **0.078*** | -1.117 | 0.84* |
| hsa-miR-143-3p | 3.56±2.35 | 3.45±1.02 | 2.45±0.83 | 2.96±1.69 | 2.157 | 0.089* | 1.404 | 0.237* |
| hsa-miR-144-3p | 0.39±1.97 | -0.64±1.4 | 0.08±0.86 | -0.48±1.65 | 1.240 | 0.622 | -1.118 | 0.8 |
| hsa-miR-144-5p | 5.65±1.93 | 4.85±1.66 | 6.28±1.15 | 4.96±1.08 | -1.553 | 0.338 | -1.083 | 0.842 |
| hsa-miR-145-5p | 3.64±0.6 | 3.15±1.13 | 3.4±0.57 | 3±0.93 | 1.183 | 0.321 | 1.112 | 0.722 |
| hsa-miR-146a-5p | 1.25±1.07 | 1.81±1.27 | 0.8±0.87 | 1.76±0.89 | 1.364 | 0.273 | 1.035 | 0.912 |
| hsa-miR-146b-5p | 7.02±0.92 | 7.32±1.58 | 7.09±1.19 | 6.47±1.24 | -1.046 | 0.882 | 1.799 | 0.159 |
| hsa-miR-148a-3p | 2.97±0.89 | 2.8±1.06 | 2.1±0.6 | 2.59±0.62 | 1.836 | 0.010 | 1.16 | 0.885* |
| hsa-miR-148b-3p | 1.85±1.27 | 2.04±1.05 | 1.41±0.7 | 1.9±0.44 | 1.358 | 0.302 | 1.106 | 0.663 |
| hsa-miR-150-5p | 2.1±1.33 | 2.01±1.45 | 2.4±1.02 | 1.6±0.98 | -1.227 | 0.548 | 1.327 | 0.428 |
| hsa-miR-151a-3p | 2.57±0.9 | 3.21±1.67 | 2.84±0.7 | 3.14±1.37 | -1.208 | 0.416 | 1.05 | 0.91 |
| hsa-miR-151a-5p | 1.52±1.1 | 2.21±1.12 | 1.47±0.6 | 1.99±1.2 | 1.035 | 0.892 | 1.163 | 0.65 |
| hsa-miR-152-3p | 3.43±0.91 | 4.06±1.08 | 3.25±0.95 | 4.28±1.59 | 1.128 | 0.651 | -1.16 | 0.704 |
| hsa-miR-154-5p | 4.95±1.65 | 5.36±1.94 | 4.59±1.39 | 5.33±0.97 | 1.281 | 0.572 | 1.017 | 0.97 |
| hsa-miR-155-5p | 6.74±1.74 | 7.63±1.73 | 7.17±1.88 | 7.42±2.11 | -1.348 | 0.840* | 1.157 | 0.299* |
| hsa-miR-15a-5p | -0.77±1.06 | -1.3±1.15 | -0.88±0.42 | -1.31±1.17 | 1.083 | 0.730 | 1.013 | 0.969 |
| hsa-miR-15b-3p | 3.86±1.11 | 3.43±0.69 | 3.9±0.52 | 3.98±1.13 | -1.028 | 0.371* | -1.46 | 0.436* |
| hsa-miR-15b-5p | 3.65±0.9 | 2.09±0.9 | 3.06±0.74 | 2.58±1.68 | 1.498 | 0.098 | -1.403 | 0.403* |
| hsa-miR-16-2-3p | 4.94±1.01 | 4.47±1.22 | 4.92±0.65 | 4.33±1.35 | 1.013 | 0.959 | 1.101 | 0.794 |
| hsa-miR-16-5p | -5.79±1 | -5.58±0.96 | -5.29±0.47 | -5.27±0.6 | -1.417 | 0.130 | -1.242 | 0.348 |
| **hsa-miR-17-5p** | 6.65±1.63 | 6.62±1.28 | 5.64±0.57 | 6.53±1.59 | **2.02** | **0.030*** | 1.066 | 0.876 |
| hsa-miR-181a-5p | 3.65±1.01 | 3.93±1.46 | 3.22±1.01 | 3.56±1.09 | 1.342 | 0.314 | 1.29 | 0.493 |
| hsa-miR-185-5p | -1.09±0.83 | -0.62±0.4 | -1.13±0.52 | -0.94±0.69 | 1.022 | 0.912 | 1.25 | 0.371* |
| hsa-miR-186-5p | 6.38±2.29 | 6.7±1.07 | 6.41±1.29 | 6.55±1.54 | -1.021 | 0.371* | 1.108 | 0.403* |
| hsa-miR-18a-5p | 2.67±1.04 | 3.28±1.23 | 2.75±1.47 | 3.02±1.34 | -1.058 | 0.977* | 1.201 | 0.618 |
| hsa-miR-18b-5p | 2.99±0.99 | 3.37±1.2 | 2.87±0.96 | 3.1±1.35 | 1.084 | 0.665* | 1.207 | 0.607 |
| hsa-miR-191-5p | 1.43±0.74 | 1.52±0.82 | 1.6±1.03 | 1.15±0.99 | -1.121 | 0.658 | 1.296 | 0.325 |
| hsa-miR-192-5p | 3.33±0.81 | 3.06±1.06 | 3.77±1.04 | 3.09±1 | -1.356 | 0.078* | -1.022 | 0.941 |
| hsa-miR-193a-5p | 9.03±1.96 | 8.2±1.58 | 8.6±1.58 | 8.88±1.23 | 1.345 | 0.563 | -1.603 | 0.252 |
| hsa-miR-194-5p | 5.36±0.85 | 4.28±1.22 | 5.6±1.18 | 4.95±1.37 | -1.177 | 0.58 | -1.593 | 0.218 |
| hsa-miR-195-5p | 5.98±1.43 | 6.63±1.61 | 6.41±0.7 | 6.37±1.28 | -1.354 | 0.089* | 1.198 | 0.885* |
| hsa-miR-197-3p | 3.99±2.15 | 3.67±0.85 | 3.5±0.86 | 4.19±2.55 | 1.4 | 0.977* | -1.428 | 0.707* |
| **hsa-miR-199a-3p** | 2.73±1.77 | 2.92±1.29 | 1.65±0.82 | 2.83±1.58 | **2.104** | **0.069** | 1.066 | 0.878 |
| hsa-miR-199a-5p | 4.83±3.25 | 4.94±2.78 | 3.38±2.43 | 3.99±2.47 | 2.722 | 0.341* | 1.929 | 0.387 |
| hsa-miR-19a-3p | -1.14±1.13 | -1.67±0.53 | -1.61±0.41 | -1.42±0.47 | 1.388 | 0.665* | -1.192 | 0.175* |
| hsa-miR-19b-3p | -2.37±0.67 | -2.52±0.6 | -2.51±0.59 | -2.16±0.64 | 1.105 | 0.665* | -1.282 | 0.157* |
| hsa-miR-200a-3p | 9.93±2.14 | 10.15±2.71 | 9.49±2.21 | 9.54±1.49 | 1.357 | 0.436* | 1.527 | 0.26* |
| **hsa-miR-200c-3p** | 9.21±2.2 | 8.93±1.99 | 7.71±1.36 | 8.59±2.04 | **2.844** | **0.053*** | 1.267 | 0.681 |
| hsa-miR-205-5p | 9.61±2.26 | 8.66±2.48 | 8.93±1.6 | 7.78±1.8 | 1.595 | 0.237* | 1.839 | 0.332 |
| hsa-miR-20a-5p | -0.69±0.76 | -0.21±0.6 | -0.62±0.43 | -0.33±0.6 | -1.052 | 0.775 | 1.084 | 0.638 |
| hsa-miR-20b-5p | 9.53±1.55 | 8.76±1.31 | 8.78±1.78 | 8.33±1.32 | 1.683 | 0.100* | 1.348 | 0.43 |
| **hsa-miR-210-3p** | 5.8±1.81 | 5.42±1.56 | 4.49±0.52 | 5.24±0.78 | **2.475** | **0.019*** | 1.133 | 0.885* |
| hsa-miR-2110 | 7.65±1.72 | 7.39±1.03 | 7.15±0.53 | 8.04±1.73 | 1.411 | 0.751* | -1.577 | 0.271 |
| hsa-miR-215-5p | 4.8±0.78 | 4.42±1 | 4.94±1.04 | 4.87±0.97 | -1.099 | 0.341* | -1.364 | 0.278 |
| hsa-miR-21-5p | -3.07±0.56 | -2.77±0.46 | -3.15±0.61 | -2.81±0.46 | 1.058 | 0.885* | 1.029 | 0.829 |
| hsa-miR-221-3p | 0.6±2.05 | 1.02±1.53 | -0.5±0.8 | 0.25±1.25 | 2.143 | 0.214* | 1.705 | 0.191 |
| hsa-miR-222-3p | 0.68±0.97 | 0.86±0.56 | 0.67±0.78 | 0.98±0.51 | 1.006 | 0.982 | -1.08 | 0.617 |
| hsa-miR-223-3p | -4.41±1.44 | -3.95±1.38 | -4.07±1.02 | -3.85±1.67 | -1.261 | 0.237* | -1.073 | 0.871 |
| hsa-miR-223-5p | 6.68±1.97 | 6.83±1.15 | 6.44±0.85 | 7.72±2.1 | 1.18 | 0.704 | -1.851 | 0.285* |
| hsa-miR-22-3p | 1.61±1.34 | 1.96±1.45 | 0.94±0.57 | 1.97±1.4 | 1.589 | 0.127 | -1.007 | 0.977* |
| **hsa-miR-22-5p** | 5.97±2.12 | 5.98±2.2 | 4.7±0.6 | 5.61±1.42 | **2.414** | **0.017*** | 1.294 | 0.751* |
| hsa-miR-23a-3p | -1.84±0.85 | -1.73±0.78 | -1.96±0.46 | -1.73±0.75 | 1.087 | 0.668 | -1.005 | 0.982 |
| hsa-miR-23b-3p | -0.3±0.99 | 0.29±0.9 | -0.25±0.76 | 0.04±0.8 | -1.037 | 0.887 | 1.191 | 0.477 |
| hsa-miR-24-3p | -2±1.25 | -1.34±1.01 | -1.76±0.8 | -1.26±1.05 | -1.180 | 0.544* | -1.061 | 0.841 |
| hsa-miR-25-3p | -0.8±0.99 | -0.53±0.99 | -0.41±0.5 | -0.34±0.59 | -1.308 | 0.237 | -1.138 | 0.583 |
| hsa-miR-26a-5p | 2.49±1.19 | 2.3±1.49 | 2.69±1.07 | 2.22±1.29 | -1.145 | 0.676 | 1.058 | 0.888 |
| hsa-miR-26b-5p | 4.33±1.82 | 3.65±0.99 | 3.96±0.97 | 4.05±1.02 | 1.291 | 0.931* | -1.325 | 0.334 |
| hsa-miR-27a-3p | 0.74±1.47 | 1.08±1.35 | 0.55±0.89 | 0.91±1 | 1.136 | 0.931* | 1.122 | 0.735 |
| hsa-miR-27b-3p | -0.21±1.64 | -0.35±1.39 | -0.84±0.69 | -0.28±1.04 | 1.546 | 0.235 | -1.051 | 0.886 |
| hsa-miR-28-3p | 4.41±0.98 | 4.69±0.74 | 4.15±0.52 | 4.63±1.15 | 1.194 | 0.431 | 1.046 | 0.665* |
| hsa-miR-28-5p | 6.56±2.55 | 5.91±1.52 | 6.09±2.41 | 5.98±2.22 | 1.38 | 0.840* | -1.053 | 0.924 |
| hsa-miR-29a-3p | 2.97±0.84 | 3.03±0.68 | 2.53±0.75 | 2.7±0.94 | 1.364 | 0.181 | 1.254 | 0.34 |
| hsa-miR-29b-3p | 4.38±1.34 | 4.57±1.06 | 3.5±0.7 | 3.65±0.72 | 1.845 | 0.055 | 1.884 | 0.046* |
| hsa-miR-29c-3p | 2.69±0.68 | 2.5±0.79 | 2.32±0.62 | 2.61±1.08 | 1.295 | 0.175 | -1.083 | 0.768 |
| hsa-miR-301a-3p | 3.87±0.95 | 3.83±1.05 | 4.4±1.46 | 3.99±1.51 | -1.446 | 0.301 | -1.12 | 0.762 |
| hsa-miR-30a-5p | 5.82±1.81 | 5.23±0.76 | 5.6±0.78 | 5.98±0.91 | 1.168 | 0.751* | -1.69 | 0.037 |
| hsa-miR-30b-5p | 2.25±0.58 | 1.73±0.94 | 2.27±0.64 | 1.76±0.65 | -1.015 | 0.931 | -1.019 | 0.937 |
| hsa-miR-30c-5p | 4.07±2.22 | 2.78±0.63 | 3.7±0.69 | 3.26±1.36 | 1.289 | 0.931* | -1.395 | 0.279 |
| hsa-miR-30d-5p | 0.61±0.86 | 0.75±0.77 | 0.52±0.4 | 0.58±0.68 | 1.065 | 0.624* | 1.119 | 0.59 |
| hsa-miR-30e-3p | 6.35±1.27 | 6.4±1.53 | 6.6±1.19 | 6.11±1.39 | -1.19 | 0.403* | 1.223 | 0.632 |
| hsa-miR-30e-5p | 1.72±1.44 | 1.81±0.61 | 1.26±0.34 | 2.22±1.26 | 1.369 | 0.840* | -1.328 | 0.322 |
| hsa-miR-320a | -1.1±0.92 | -0.69±0.71 | -0.55±0.78 | -0.46±0.93 | -1.464 | 0.128 | -1.177 | 0.493 |
| hsa-miR-320b | 1.14±0.93 | 1.31±0.68 | 1.83±0.75 | 1.68±0.95 | -1.607 | 0.06 | -1.291 | 0.287 |
| hsa-miR-320c | 3.11±1.62 | 3.56±0.8 | 3.29±0.84 | 4.09±1.6 | -1.13 | 0.741 | -1.445 | 0.315 |
| hsa-miR-320d | 3.83±1.01 | 3.87±0.66 | 4.19±0.72 | 4.07±0.99 | -1.283 | 0.327 | -1.147 | 0.569 |
| hsa-miR-324-3p | 4.31±0.34 | 4.86±1.36 | 4.37±1.78 | 4.83±1.67 | -1.043 | 0.017* | 1.025 | 0.47* |
| hsa-miR-324-5p | 4.93±2.15 | 4.58±1.25 | 4.04±1.4 | 5.15±2.85 | 1.862 | 0.238 | -1.487 | 0.531 |
| hsa-miR-32-5p | 3.19±1.06 | 2.37±0.97 | 2.83±0.61 | 2.36±1.01 | 1.278 | 0.328 | 1.006 | 0.983 |
| **hsa-miR-326** | 7.27±2.9 | 7.65±2.25 | 5.3±1.39 | 6.69±2.67 | **3.927** | **0.045** | 1.945 | 0.141* |
| hsa-miR-328-3p | 2.87±1.87 | 3.68±1.18 | 3.21±1 | 3.23±1.19 | -1.266 | 0.584 | 1.371 | 0.356 |
| hsa-miR-331-3p | 4.56±1.14 | 5.18±1.62 | 4.2±1.31 | 4.32±1.58 | 1.278 | 0.403* | 1.809 | 0.203 |
| hsa-miR-335-3p | 7.26±2.87 | 7.32±1.95 | 6.82±1.92 | 7.32±2.71 | 1.354 | 0.885* | -1.006 | 0.285* |
| hsa-miR-335-5p | 7.37±1.49 | 6.83±1.69 | 7.19±1.48 | 6.41±0.98 | 1.135 | 0.766 | 1.341 | 0.46 |
| hsa-miR-338-3p | 5.55±1.37 | 5.98±1.44 | 4.63±1.09 | 5.17±1.08 | 1.891 | 0.082 | 1.76 | 0.131 |
| hsa-miR-339-3p | 4.76±2.1 | 6.3±2.88 | 4.3±0.66 | 5.73±2.92 | 1.38 | 0.751* | 1.479 | 0.436* |
| hsa-miR-339-5p | 5.34±2.72 | 4.9±2 | 4.12±1.96 | 4.61±2.09 | 2.33 | 0.112* | 1.222 | 0.732 |
| **hsa-miR-33a-5p** | 6.5±3.25 | 5.73±1.92 | 4.18±1.18 | 5.34±2.05 | **5.012** | **0.035*** | 1.307 | 0.583* |
| hsa-miR-342-3p | 3±0.71 | 3.47±0.75 | 3.15±0.61 | 2.95±0.74 | -1.114 | 0.57 | 1.439 | 0.097 |
| hsa-miR-34a-5p | 8.04±1.95 | 6.46±1.4 | 7.59±1.26 | 6.88±2.01 | 1.371 | 0.504 | -1.34 | 0.341* |
| hsa-miR-361-5p | 1.88±1.04 | 2.7±0.94 | 2.06±0.62 | 2.13±0.68 | -1.13 | 0.618 | 1.483 | 0.103 |
| hsa-miR-362-3p | 5.9±0.77 | 5.8±1.41 | 5.65±0.63 | 5.78±0.57 | 1.185 | 0.406 | 1.018 | 0.977* |
| hsa-miR-363-3p | 3.2±0.71 | 2.56±1.02 | 3.21±0.48 | 2.68±1.04 | -1.009 | 0.96 | -1.086 | 0.78 |
| hsa-miR-365a-3p | 9.89±2.45 | 9.56±2.03 | 9.28±2.22 | 8.67±2.44 | 1.535 | 0.524 | 1.854 | 0.341 |
| hsa-miR-374a-5p | 6.58±1.73 | 5.66±1.59 | 6.03±1.04 | 5.16±0.63 | 1.465 | 0.354 | 1.415 | 0.32 |
| hsa-miR-374b-5p | 4.68±0.99 | 4.9±1.82 | 4.6±1.3 | 4.44±2.3 | 1.056 | 0.931* | 1.38 | 0.312* |
| hsa-miR-375 | 5.89±1.74 | 6.02±2.4 | 6.2±1.46 | 6.18±1.75 | -1.241 | 0.64 | -1.116 | 0.855 |
| hsa-miR-376a-3p | 4.82±2.83 | 4.93±1.99 | 4.56±1.69 | 5.13±1.84 | 1.202 | 0.751* | -1.15 | 0.84* |
| hsa-miR-376c-3p | 4.39±2.97 | 4.67±1.75 | 4.08±1.59 | 4.69±2.19 | 1.242 | 0.751 | -1.016 | 0.665* |
| hsa-miR-382-5p | 4.79±2.24 | 5.38±1.8 | 5.25±1.85 | 6.05±2.26 | -1.373 | 0.194* | -1.594 | 0.429 |
| hsa-miR-409-3p | 3.97±2.75 | 6.16±3.05 | 4.59±1.46 | 5.09±1.87 | -1.533 | 0.069* | 2.103 | 0.311 |
| hsa-miR-421 | 5.92±0.71 | 6.94±1.22 | 6.24±1.62 | 6.48±2.35 | -1.253 | 0.931* | 1.377 | 0.069* |
| hsa-miR-423-3p | 2.54±1.13 | 2.62±1.08 | 1.8±0.99 | 2.16±0.84 | 1.666 | 0.103 | 1.38 | 0.252 |
| hsa-miR-423-5p | 2.51±1.12 | 2.46±0.97 | 1.97±0.67 | 2.36±0.68 | 1.451 | 0.061* | 1.071 | 0.776 |
| hsa-miR-424-5p | 4.66±1.15 | 4.27±1.31 | 4.15±0.76 | 4.52±1.65 | 1.427 | 0.212 | -1.188 | 0.688 |
| hsa-miR-425-3p | 4.69±0.65 | 4.91±0.75 | 4.58±0.83 | 5.01±1.14 | 1.079 | 0.436* | -1.073 | 0.8 |
| hsa-miR-425-5p | 3.29±2.63 | 2.26±0.91 | 1.81±0.64 | 1.86±0.76 | 2.778 | 0.126* | 1.315 | 0.261 |
| hsa-miR-451a | -4.97±0.92 | -5.07±1.19 | -5.27±0.92 | -4.9±1.69 | 1.235 | 0.427 | -1.12 | 0.787 |
| hsa-miR-454-3p | 8.38±2.22 | 6.21±0.96 | 7.19±1.98 | 6.05±0.66 | 2.272 | 0.181 | 1.114 | 0.649 |
| hsa-miR-483-5p | 7.78±1.14 | 7.81±2.6 | 8.78±1.72 | 7.5±2.38 | -1.998 | 0.108 | 1.241 | 0.763 |
| hsa-miR-484 | 1.37±0.77 | 1.93±0.69 | 1.38±0.68 | 1.68±0.61 | -1.011 | 0.958 | 1.183 | 0.373 |
| hsa-miR-485-3p | 6.16±1.53 | 6.99±1.51 | 6.78±1.4 | 6.36±1.25 | -1.532 | 0.316 | 1.542 | 0.283 |
| hsa-miR-486-5p | -1.05±1.1 | -0.84±0.99 | -0.41±0.95 | -0.77±0.87 | -1.562 | 0.14 | -1.053 | 0.846 |
| hsa-miR-495-3p | 6.93±2.51 | 7.26±2.38 | 6.82±2.09 | 6.98±1.55 | 1.081 | 0.795* | 1.218 | 0.732 |
| **hsa-miR-497-5p** | 8.8±2.08 | 8.24±2.89 | 7.13±1.15 | 7.98±2.41 | **3.185** | **0.024** | 1.196 | 0.665* |
| **hsa-miR-501-3p** | 9.21±3.09 | 6.9±1.37 | 7.33±1.11 | 7.83±1.72 | **3.687** | **0.06** | -1.895 | 0.161 |
| hsa-miR-502-3p | 6.4±0.78 | 6.07±1.62 | 6.36±0.82 | 5.9±1.33 | 1.03 | 0.898 | 1.12 | 0.544* |
| hsa-miR-505-3p | 4.74±0.68 | 5.26±1.05 | 4.57±0.89 | 5.33±0.52 | 1.13 | 0.592 | -1.051 | 0.835 |
| hsa-miR-532-3p | 4.89±0.41 | 5.22±0.74 | 5.51±0.62 | 5.07±0.88 | -1.541 | 0.008 | 1.108 | 0.84* |
| hsa-miR-532-5p | 5.2±0.69 | 5.05±1.06 | 5.58±1.37 | 5.5±1.55 | -1.302 | 0.399 | -1.364 | 0.341* |
| hsa-miR-543 | 6.66±2.12 | 7.69±2.05 | 7.07±1.48 | 7.43±1.43 | -1.323 | 0.260* | 1.199 | 0.72 |
| hsa-miR-574-3p | 4.22±2.58 | 4.01±1.34 | 3.42±0.5 | 4.14±2.48 | 1.745 | 0.470* | -1.099 | 0.583* |
| hsa-miR-584-5p | 5.22±1.13 | 5.73±1.32 | 5.22±0.77 | 5.53±1.13 | -1.004 | 0.988 | 1.149 | 0.694 |
| hsa-miR-590-5p | 2.17±0.84 | 2.17±0.58 | 2.54±0.59 | 2.59±0.66 | -1.293 | 0.157* | -1.345 | 0.104 |
| hsa-miR-629-5p | 7.3±2.13 | 6.56±2.64 | 7.11±1.99 | 7.78±1.9 | 1.142 | 0.795* | -2.334 | 0.206 |
| hsa-miR-652-3p | 2.74±1.57 | 3.92±2.16 | 2.11±0.87 | 3.31±2.11 | 1.551 | 0.234 | 1.535 | 0.485 |
| hsa-miR-660-5p | 3.38±0.92 | 3.53±2.05 | 3.35±0.71 | 2.99±1.21 | 1.02 | 0.934 | 1.456 | 0.438 |
| hsa-miR-7-1-3p | 5.99±1.58 | 6.82±1.4 | 6.19±0.88 | 6.76±1.68 | -1.144 | 0.714 | 1.048 | 0.916 |
| hsa-miR-7-5p | 7.36±1.28 | 7.24±1.54 | 7.74±1.35 | 7.03±1.09 | -1.301 | 0.486 | 1.158 | 0.701 |
| hsa-miR-766-3p | 5.43±1.42 | 5.68±1.87 | 4.97±1.01 | 4.71±1.38 | 1.368 | 0.544* | 1.958 | 0.162 |
| **hsa-miR-874-3p** | 8.24±1.96 | 7.65±1.95 | 6.35±0.9 | 6.82±1.3 | **3.722** | **0.006** | 1.776 | 0.233 |
| hsa-miR-877-5p | 6.6±1.31 | 7.39±1.63 | 7.9±2.14 | 7.06±1.24 | -2.47 | 0.285* | 1.257 | 0.581 |
| hsa-miR-885-5p | 8.38±1.44 | 7.71±2.39 | 7.96±2.21 | 7.37±1.88 | 1.332 | 0.977* | 1.268 | 0.7 |
| hsa-miR-92a-3p | -1.43±0.8 | -1.92±0.78 | -1.42±0.61 | -1.84±0.64 | -1.005 | 0.840* | -1.06 | 0.795* |
| hsa-miR-92b-3p | 8.24±1.32 | 8.19±1.3 | 8.65±1.3 | 8.65±1.64 | -1.326 | 0.454 | -1.377 | 0.453 |
| hsa-miR-93-3p | 5.19±1.39 | 5.53±0.89 | 5.67±1.74 | 6.43±2.78 | -1.39 | 0.312* | -1.87 | 0.707* |
| hsa-miR-93-5p | -0.06±0.6 | 0.25±0.47 | -0.3±0.53 | -0.1±0.67 | 1.185 | 0.301 | 1.28 | 0.147 |
| hsa-miR-99a-5p | 6.17±1.1 | 5.65±1.14 | 5.59±0.97 | 5.36±1.16 | 1.5 | 0.182 | 1.223 | 0.541 |
| hsa-miR-99b-5p | 5.61±1.77 | 5.12±1.1 | 5.19±0.65 | 5.78±2.35 | 1.345 | 0.441 | -1.581 | 0.751* |
| mmu-miR-378a-3p | 3.87±1.19 | 3.2±0.85 | 3.74±0.6 | 3.23±0.73 | 1.09 | 0.624* | -1.02 | 0.93 |

Fold change was calculated between PIR and GIR groups using the comparative CT-method (=2 ^ – ((average ∆CT miRNA of interest in PIR-group ) – (average ∆CT miRNA of interest in GIR-group)). Differences between groups were tested using students t-test or Mann-Whitney, based on the results of the KS-normality test; p-values for non-normal distributions are indicated with an asterisk (*). MicroRNAs that are considered different between study groups are bold. A negative (positive) FC means a higher (lower) level of the respective microRNA in the PIR group compared to GIR group. Grey shaded microRNAs indicated microRNAs selected for further analysis.

Abbreviations: D0, Pre-ART; M12, Month 12 after ART initiation; PIR, Poor immune recovery; GIR, Good immune recovery

**Table S4. Pathways and targets of hsa-miR-17-5p**

| **Pathway** | **Hits** | **Expected hits** | **P-value** | **Targets** |
| --- | --- | --- | --- | --- |
| Cyclin D associated events in G1 | 8 | 0.826 | 8.10e-5 | CCND1, CCND2,CDKN1A,E2F1,PPP2R2A,RB1,RBL1,RBL2 |
| G1 Phase | 8 | 0.826 | 8.10e-5 | CCND1, CCND2,CDKN1A,E2F1,PPP2R2A,RB1,RBL1,RBL2 |
| Transcriptional regulation by RUNX3 | 9 | 1.15 | 8.10e-5 | BCL2L11, CCND1,CDKN1A,KAT2B,MDM2,MYC,RUNX1,SMAD4,SMURF1 |
| Cyclin E associated events during G1/S transition | 7 | 0.826 | 4.29e-4 | CCND1, CDKN1A,E2F1,MYC,RB1,RBL2,WEE1 |
| Mitotic G1-G1/S phases | 10 | 1.89 | 4.29e-4 | CCND1, CCND2,CDKN1A,E2F1,MYC,PPP2R2A,RB1,RBL1,RBL2,WEE1 |
| S Phase | 9 | 1.45 | 4.29e-4 | CCND1, CDKN1A,E2F1,MYC,RAD21,RB1,RBL2,UBE2C,WEE1 |
| Cyclin A:Cdk2-associated events at S phase entry | 7 | 0.855 | 4.32e-4 | CCND1, CDKN1A,E2F1,MYC,RB1,RBL2,WEE1 |
| G1/S Transition | 8 | 1.42 | 0.002 | CCND1, CDKN1A,E2F1,MYC,RB1,RBL1,RBL2,WEE1 |
| Cell Cycle | 14 | 4.57 | 0.003 | BRCA2, CCND1,CCND2,CDKN1A,E2F1,MDM2,MYC  ,PPP2R2A,RAD21,RB1,RBL1,RBL2,UBE2C,WEE1 |
| Interleukin-4 and Interleukin-13 signaling | 10 | 2.54 | 0.004 | BCL2, CCND1,CDKN1A,HIF1A,JAK1,MMP2,MYC,STAT3,TNF,VEGFA |
| Signaling by TGF-beta family members | 8 | 1.71 | 0.005 | BMP2, BMPR2,MYC,RBL1,SMAD4,SMURF1,TGFBR2,ZFYVE9 |
| Transcription of E2F targets under negative control by DREAM complex | 4 | 0.354 | 0.005 | E2F1, MYC,RBL1,RBL2 |
| Transcriptional regulation by RUNX2 | 8 | 1.74 | 0.005 | BMP2, CCND1,CDKN1A,RB1,RUNX1,SMAD4,SMURF1,YES1 |
| Cell Cycle, Mitotic | 12 | 3.92 | 0.006 | CCND1, CCND2,CDKN1A,E2F1,MYC,PPP2R2A,RAD21,RB1,RBL1,RBL2,UBE2C,WEE1 |
| Signaling by BMP | 4 | 0.413 | 0.008 | BMP2, BMPR2,SMAD4,SMURF1 |
| Estrogen-dependent gene expression | 7 | 1.56 | 0.010 | BCL2, CCND1,KAT2B,MYC,NCOA3,RAD21,RUNX1 |
| Loss of Function of SMAD2/3 in Cancer | 3 | 0.206 | 0.010 | SMAD4, TGFBR2,ZFYVE9 |
| Signaling by TGF-beta Receptor Complex in Cancer | 3 | 0.206 | 0.010 | SMAD4, TGFBR2,ZFYVE9 |
| Transcriptional regulation of granulopoiesis | 5 | 0.796 | 0.011 | CDKN1A, E2F1,MYC,RUNX1,STAT3 |
| BH3-only proteins associate with and inactivate anti-apoptotic BCL-2 members | 3 | 0.236 | 0.012 | BCL2, BCL2L11,STAT3 |
| RNA Polymerase II Transcription | 22 | 11.45 | 0.012 | BCL2L11, BMP2,CCND1,CCND2,CDKN1A,E2F1,IGFBP3,KAT2B,MDM2,MYC,NABP1,PTEN,RB1,RBL1,RBL2,RUNX1,SMAD4,SMURF1,TCF3,TP53INP1,VEGFA,YES1 |
| Regulation of gene expression by Hypoxia-inducible Factor | 3 | 0.236 | 0.012 | EPAS1, HIF1A,VEGFA |
| TGF-beta receptor signaling activates SMADs | 4 | 0.501 | 0.012 | SMAD4, SMURF1,TGFBR2,ZFYVE9 |
| Gene expression (Transcription) | 23 | 12.33 | 0.014 | BCL2L11, BMP2,CCND1,CCND2,CDKN1A,DNMT1,E2F1,IGFBP3,KAT2B,MDM2,MYC,NABP1,PTEN,RB1,RBL1,RBL2,RUNX1,SMAD4,SMURF1,TCF3,TP53INP1,VEGFA,YES1 |
| Signaling by TGF-beta Receptor Complex | 6 | 1.33 | 0.016 | MYC, RBL1,SMAD4,SMURF1,TGFBR2,ZFYVE9 |
| G0 and Early G1 | 4 | 0.590 | 0.019 | E2F1, MYC,RBL1,RBL2 |
| Generic Transcription Pathway | 21 | 11.18 | 0.019 | BCL2L11, BMP2,CCND1,CCND2,CDKN1A,E2F1,IGFBP3,KAT2B,MDM2,MYC,PTEN,RB1,RBL1,RBL2,RUNX1,SMAD4,SMURF1,TCF3,TP53INP1,VEGFA,YES1 |
| PTK6 Expression | 2 | 0.088 | 0.019 | EPAS1, HIF1A |
| Transcription of E2F targets under negative control by p107 (RBL1) and p130 (RBL2) in complex with HDAC1 | 3 | 0.295 | 0.019 | E2F1, RBL1,RBL2 |
| Developmental Biology | 18 | 9.35 | 0.029 | CDKN1A, E2F1,EGR2,EPAS1,KAT2B,MEF2D,MMP2,MYC,NCOA3,PDLIM7,PKNOX1,RUNX1,SMAD4,STAT3,TCF3,TNF,VLDLR,YES1 |
| Interleukin-27 signaling | 2 | 0.118 | 0.035 | JAK1, STAT3 |
| ESR-mediated signaling | 8 | 2.77 | 0.038 | BCL2, CCND1,KAT2B,MMP2,MYC,NCOA3,RAD21,RUNX1 |
| Pre-NOTCH Transcription and Translation | 4 | 0.737 | 0.038 | CCND1, E2F1,KAT2B,RUNX1 |
| Cellular Senescence | 7 | 2.24 | 0.040 | CDKN1A, E2F1,MAPK9,MDM2,RB1,STAT3,UBE2C |
| TP53 Regulates Transcription of Cell Cycle Genes | 4 | 0.767 | 0.040 | CDKN1A, E2F1,RBL1,RBL2 |
| Cellular responses to stress | 10 | 4.10 | 0.041 | CDKN1A, E2F1,EPAS1,HIF1A,MAPK9,MDM2,RB1,STAT3,UBE2C,VEGFA |
| Downregulation of TGF-beta receptor signaling | 3 | 0.413 | 0.041 | SMURF1, TGFBR2,ZFYVE9 |
| Regulation of RUNX1 Expression and Activity | 3 | 0.413 | 0.041 | CCND1, CCND2,RUNX1 |
| Formation of Senescence-Associated Heterochromatin Foci (SAHF) | 2 | 0.147 | 0.042 | CDKN1A, RB1 |
| Inhibition of replication initiation of damaged DNA by RB1/E2F1 | 2 | 0.147 | 0.042 | E2F1, RB1 |
| RUNX3 regulates BCL2L11 (BIM) transcription | 2 | 0.147 | 0.042 | BCL2L11, SMAD4 |
| TFAP2 (AP-2) family regulates transcription of cell cycle factors | 2 | 0.147 | 0.042 | CDKN1A, MYC |
| FOXO-mediated transcription of cell cycle genes | 3 | 0.442 | 0.043 | CDKN1A, RBL2,SMAD4 |
| Signal Transduction | 33 | 22.77 | 0.043 | APP, BCL2,BCL2L11,BMP2,BMPR2,CCL1,CCND1,CDKN1A,E2F1,EPAS1,HIF1A,JAK1,KAT2B,LDLR,MDM2,MMP2,MYC,NCOA3,PHLPP1,PTEN,PTPRO,RAD21,RBL1,RUNX1,SMAD4,SMURF1,SOCS6,STAT3,TGFBR2,TNF,VEGFA,YES1,ZFYVE9 |
| Cellular responses to external stimuli | 11 | 5.10 | 0.050 | CDKN1A, E2F1,EPAS1,HIF1A,MAPK9,MDM2,MFN2,RB1,STAT3,UBE2C,VEGFA |
| FOXO-mediated transcription | 5 | 1.39 | 0.050 | BCL2L11, CDKN1A,KAT2B,RBL2,SMAD4 |
| Interleukin-9 signaling | 2 | 0.177 | 0.050 | JAK1, STAT3 |
| Loss of Function of TGFBR1 in Cancer | 2 | 0.177 | 0.050 | TGFBR2, ZFYVE9 |
| Pre-NOTCH Expression and Processing | 4 | 0.914 | 0.050 | CCND1, E2F1,KAT2B,RUNX1 |
| RUNX3 regulates CDKN1A transcription | 2 | 0.177 | 0.050 | CDKN1A, SMAD4 |
| SMAD2/3 Phosphorylation Motif Mutants in Cancer | 2 | 0.177 | 0.050 | TGFBR2, ZFYVE9 |
| Signaling by Nuclear Receptors | 8 | 3.10 | 0.050 | BCL2, CCND1,KAT2B,MMP2,MYC,NCOA3,RAD21,RUNX1 |
| TGFBR1 KD Mutants in Cancer | 2 | 0.177 | 0.050 | TGFBR2, ZFYVE9 |

**Table S5.** **Pathways and targets of hsa-miR-199a-3p**

| **Pathway** | **Hits** | **Expected hits** | **P-value** | **Targets** |
| --- | --- | --- | --- | --- |
| Signaling by Receptor Tyrosine Kinases | 11 | 1.781 | 2.28e-5 | APOE, FLT1,HGF,IGF1,ITGA3,KDR,MAPK1,MET,MTOR,VEGFA,YAP1 |
| VEGF binds to VEGFR leading to receptor dimerization | 3 | 0.042 | 1.46e-4 | FLT1, KDR,VEGFA |
| VEGF ligand-receptor interactions | 3 | 0.042 | 1.46e-4 | FLT1, KDR,VEGFA |
| MET activates PTK2 signaling | 3 | 0.076 | 8.99e-4 | HGF, ITGA3,MET |
| MET Receptor Activation | 2 | 0.017 | 0.001 | HGF, MET |
| MET activates STAT3 | 2 | 0.025 | 0.002 | HGF, MET |
| MET interacts with TNS proteins | 2 | 0.025 | 0.002 | HGF, MET |
| MET promotes cell motility | 3 | 0.144 | 0.003 | HGF, ITGA3,MET |
| Neurophilin interactions with VEGF and VEGFR | 2 | 0.034 | 0.004 | FLT1, KDR |
| MET activates PTPN11 | 2 | 0.042 | 0.006 | HGF, MET |
| Gene expression (Transcription) | 10 | 3.545 | 0.008 | APOE, CDK7,MAPK1,MET,MTOR,SMARCA2,STK11,TFAM,VEGFA,YAP1 |
| MET activates PI3K/AKT signaling | 2 | 0.051 | 0.008 | HGF, MET |
| MET receptor recycling | 2 | 0.059 | 0.009 | HGF, MET |
| MET activates RAS signaling | 2 | 0.068 | 0.010 | HGF, MET |
| Signaling by VEGF | 4 | 0.534 | 0.010 | FLT1, KDR,MTOR,VEGFA |
| Energy dependent regulation of mTOR by LKB1-AMPK | 2 | 0.076 | 0.011 | MTOR, STK11 |
| Generic Transcription Pathway | 9 | 3.214 | 0.011 | APOE, CDK7,MAPK1,MET,MTOR,SMARCA2,STK11,VEGFA,YAP1 |
| Integrin cell surface interactions | 3 | 0.271 | 0.011 | CD44, ITGA3,KDR |
| MET activates RAP1 and RAC1 | 2 | 0.085 | 0.011 | HGF, MET |
| Negative regulation of MET activity | 2 | 0.085 | 0.011 | HGF, MET |
| RNA Polymerase II Transcription | 9 | 3.291 | 0.011 | APOE, CDK7,MAPK1,MET,MTOR,SMARCA2,STK11,VEGFA,YAP1 |
| Signaling by MET | 3 | 0.288 | 0.011 | HGF, ITGA3,MET |
| Signal Transduction | 13 | 6.547 | 0.015 | APOE, CAV2,FLT1,HGF,IGF1,ITGA3,KDR,MAPK1,MET,MTOR,STK11,VEGFA,YAP1 |
| Hemostasis | 6 | 1.654 | 0.016 | CD44, HGF,IGF1,ITGA3,MAPK1,VEGFA |
| Platelet degranulation | 3 | 0.365 | 0.018 | HGF, IGF1,VEGFA |
| Response to elevated platelet cytosolic Ca2+ | 3 | 0.382 | 0.018 | HGF, IGF1,VEGFA |
| VEGFR2 mediated cell proliferation | 2 | 0.119 | 0.018 | KDR, VEGFA |
| Platelet activation, signaling and aggregation | 4 | 0.823 | 0.023 | HGF, IGF1,MAPK1,VEGFA |
| mTOR signalling | 2 | 0.136 | 0.023 | MTOR, STK11 |
| Nuclear signaling by ERBB4 | 2 | 0.161 | 0.029 | APOE, YAP1 |
| RUNX2 regulates osteoblast differentiation | 2 | 0.161 | 0.029 | MAPK1, YAP1 |
| Transcriptional regulation by the AP-2 (TFAP2) family of transcription factors | 2 | 0.170 | 0.031 | APOE, VEGFA |
| VEGFA-VEGFR2 Pathway | 3 | 0.492 | 0.031 | KDR, MTOR,VEGFA |
| PI5P, PP2A and IER3 Regulate PI3K/AKT Signaling | 3 | 0.526 | 0.035 | HGF, MAPK1,MET |
| RUNX1 regulates transcription of genes involved in differentiation of HSCs | 2 | 0.187 | 0.035 | CDK7, YAP1 |
| RUNX2 regulates bone development | 2 | 0.195 | 0.036 | MAPK1, YAP1 |
| Negative regulation of the PI3K/AKT network | 3 | 0.577 | 0.041 | HGF, MAPK1,MET |
| PI3K/AKT Signaling in Cancer | 3 | 0.577 | 0.041 | HGF, MET,MTOR |
| Cellular response to heat stress | 2 | 0.221 | 0.042 | MAPK1, MTOR |
| PIP3 activates AKT signaling | 4 | 1.128 | 0.048 | HGF, MAPK1,MET,MTOR |
| Transcriptional regulation by RUNX1 | 3 | 0.628 | 0.048 | CDK7, SMARCA2,YAP1 |

**Table S6.**  **Pathways and targets of hsa-miR-200c-3p**

| **Pathway** | **Hits** | **Expected hits** | **P-value** | **Targets** |
| --- | --- | --- | --- | --- |
| Signal Transduction | 43 | 25.33 | 0.011 | ACVR2B, ADAM12,BCL2,BMI1,BTC,CDK2,CRKL,DLC1,DUSP1,E2F3,EDNRA,ELMO2,ERBIN,FLT1,FN1,FOXO1,IKBKB,KDR,KRAS,LEPR,LPAR1,MYB,MYLK,NCAM1,NOS3,NOTCH1,NTF3,NTRK2,PIN1,PRKCZ,PTEN,RHOA,RNF2,ROCK2,RPS6KB1,SEPTIN7,SHC1,SP1,SUZ12,TCF7L1,VEGFA,XIAP,ZNF217 |
| SUMOylation of DNA methylation proteins | 4 | 0.295 | 0.014 | BMI1, DNMT3A,DNMT3B,RNF2 |
| Signaling by VEGF | 9 | 2.067 | 0.014 | ELMO2, FLT1,KDR,KRAS,NOS3,PRKCZ,RHOA,ROCK2,VEGFA |
| VEGF binds to VEGFR leading to receptor dimerization | 3 | 0.164 | 0.017 | FLT1, KDR,VEGFA |
| VEGF ligand-receptor interactions | 3 | 0.164 | 0.017 | FLT1, KDR,VEGFA |
| VEGFA-VEGFR2 Pathway | 8 | 1.903 | 0.021 | ELMO2, KDR,KRAS,NOS3,PRKCZ,RHOA,ROCK2,VEGFA |
| NTF3 activates NTRK2 (TRKB) signaling | 2 | 0.066 | 0.028 | NTF3, NTRK2 |
| Regulation of PTEN localization | 2 | 0.066 | 0.028 | PTEN, XIAP |
| Signaling by Receptor Tyrosine Kinases | 16 | 6.892 | 0.028 | ADAM12, BTC,CRKL,ELMO2,ERBIN,FLT1,KDR,KRAS,NOS3,NTF3,NTRK2,PRKCZ,RHOA,ROCK2,SHC1,VEGFA |
| VEGFR2 mediated cell proliferation | 4 | 0.459 | 0.028 | KDR, KRAS,PRKCZ,VEGFA |
| Activated NTRK2 signals through RAS | 3 | 0.263 | 0.037 | KRAS, NTRK2,SHC1 |
| Activated NTRK3 signals through RAS | 3 | 0.263 | 0.037 | KRAS, NTF3,SHC1 |
| Cellular Senescence | 8 | 2.494 | 0.048 | BMI1, CCNE2,CDK2,E2F3,ETS1,RNF2,SP1,SUZ12 |
| ESR-mediated signaling | 9 | 3.085 | 0.048 | BCL2, BTC,KRAS,MYB,NOS3,PRKCZ,SHC1,SP1,ZNF217 |
| Extra-nuclear estrogen signaling | 6 | 1.51 | 0.048 | BCL2, BTC,KRAS,NOS3,PRKCZ,SHC1 |
| Invadopodia formation | 2 | 0.098 | 0.048 | ADAM12, SH3PXD2A |
| SHC1 events in ERBB4 signaling | 3 | 0.328 | 0.048 | BTC, KRAS,SHC1 |
| Signaling by ERBB2 | 5 | 1.05 | 0.048 | BTC, ERBIN,KRAS,RHOA,SHC1 |
| Signaling by NTRK2 (TRKB) | 4 | 0.624 | 0.048 | KRAS, NTF3,NTRK2,SHC1 |

**Table S7. Expression of target genes of miR-17-5p in CD4+ T-cells**

| Gene (probeset) | P.Value | logFC | Gene.title |
| --- | --- | --- | --- |
| ABCA1(203504_s_at) | 0.246721 | -0.402 | ATP binding cassette subfamily A member 1 |
| ABCA1(203505_at) | 0.496861 | -0.147 | ATP binding cassette subfamily A member 1 |
| ABCA1(216066_at) | 0.931381 | 0.0753 | ATP binding cassette subfamily A member 1 |
| ADAR | 0.060417 | -0.34 | adenosine deaminase, RNA specific |
| APP(200602_at) | 0.046885 | 0.82 | amyloid beta precursor protein |
| APP(211277_x_at) | 0.22445 | -0.463 | amyloid beta precursor protein |
| APP(214953_s_at) | 0.658645 | 0.169 | amyloid beta precursor protein |
| BCL2(203684_s_at) | 0.113466 | -1.14 | BCL2, apoptosis regulator |
| BCL2(203685_at) | 0.23601 | -0.264 | BCL2, apoptosis regulator |
| BCL2(207004_at) | 0.499308 | 0.182 | BCL2, apoptosis regulator |
| BCL2(207005_s_at) | 0.789358 | -0.0825 | BCL2, apoptosis regulator |
| BCL2L11(208536_s_at) | 0.718563 | -0.301 | BCL2 like 11 |
| BCL2L11(222343_at) | 0.801558 | 0.136 | BCL2 like 11 |
| BMP2(205289_at) | 0.378165 | 0.369 | bone morphogenetic protein 2 |
| BMP2(205290_s_at) | 0.685771 | 0.251 | bone morphogenetic protein 2 |
| BMPR2(210214_s_at) | 0.473473 | 0.232 | bone morphogenetic protein receptor type 2 |
| BMPR2(209920_at) | 0.64852 | 0.268 | bone morphogenetic protein receptor type 2 |
| BRCA2(208368_s_at) | 0.954379 | 0.0699 | BRCA2, DNA repair associated |
| BRCA2(214727_at) | 0.988272 | 0.00534 | BRCA2, DNA repair associated |
| CCL1 | 0.873591 | 0.0434 | C-C motif chemokine ligand 1 |
| CCND1(208712_at) | 0.20759 | -0.637 | cyclin D1 |
| CCND1(208711_s_at) | 0.473302 | 0.438 | cyclin D1 |
| CCND2(200952_s_at) | 0.317042 | -0.336 | cyclin D2 |
| CCND2(200953_s_at) | 0.517434 | 0.113 | cyclin D2 |
| CCND2(200951_s_at) | 0.903075 | -0.0361 | cyclin D2 |
| CDKN1A | 0.021669 | 0.724 | cyclin dependent kinase inhibitor 1A |
| CLOCK(217563_at) | 0.123883 | -0.58 | clock circadian regulator |
| CLOCK(204980_at) | 0.521672 | 0.156 | clock circadian regulator |
| CLU(208791_at) | 0.448003 | 1.27 | clusterin |
| CLU(208792_s_at) | 0.448264 | 0.817 | clusterin |
| CLU(222043_at) | 0.515869 | 0.411 | clusterin |
| CYP7B1 | 0.191677 | -0.69 | cytochrome P450 family 7 subfamily B member 1 |
| DAPK3 | NA | NA |  |
| DNAJC27 | NA | NA |  |
| DNMT1 | 0.998462 | -0.00027 | DNA (cytosine-5-)-methyltransferase 1 |
| E2F1(2028_s_at) | 0.105589 | -0.595 | E2F transcription factor 1 |
| E2F1(204947_at) | 0.137902 | -0.63 | E2F transcription factor 1 |
| EGR2 | 0.034307 | 0.812 | early growth response 2 |
| EPAS1(200879_s_at) | 0.35695 | -0.55 | endothelial PAS domain protein 1 |
| EPAS1(200878_at) | 0.737094 | -0.235 | endothelial PAS domain protein 1 |
| ETV1(217061_s_at) | 0.1692 | -0.644 | ETS variant 1 |
| ETV1(221911_at) | 0.175638 | -1.02 | ETS variant 1 |
| ETV1(217053_x_at) | 0.318506 | -0.319 | ETS variant 1 |
| ETV1(221910_at) | 0.353333 | -0.512 | ETS variant 1 |
| ETV1(206501_x_at) | 0.412081 | -0.172 | ETS variant 1 |
| FBXO31 | 0.561809 | -0.232 | F-box protein 31 |
| GPR137B | 0.967806 | -0.0065 | G protein-coupled receptor 137B |
| HBP1(209102_s_at) | 0.804359 | 0.0621 | HMG-box transcription factor 1 |
| HBP1(207361_at) | 0.934617 | 0.019 | HMG-box transcription factor 1 |
| HIF1A | 0.88746 | -0.0288 | hypoxia inducible factor 1 alpha subunit |
| HSPB2 | 0.165657 | 0.535 | heat shock protein family B (small) member 2 |
| IGFBP3(212143_s_at) | 0.052002 | -0.899 | insulin like growth factor binding protein 3 |
| IGFBP3(210095_s_at) | 0.354467 | -0.398 | insulin like growth factor binding protein 3 |
| ITGB8(211488_s_at) | 0.376338 | -0.281 | integrin subunit beta 8 |
| ITGB8(205816_at) | 0.889532 | -0.079 | integrin subunit beta 8 |
| JAK1 | 0.299336 | 0.184 | Janus kinase 1 |
| KAT2B | 0.883972 | -0.0316 | lysine acetyltransferase 2B |
| LDLR(217103_at) | 0.029989 | -0.846 | low density lipoprotein receptor |
| LDLR(202068_s_at) | 0.136757 | -0.355 | low density lipoprotein receptor |
| LDLR(202067_s_at) | 0.352003 | -0.386 | low density lipoprotein receptor |
| LDLR(217173_s_at) | 0.740589 | -0.0925 | low density lipoprotein receptor |
| LDLR(217005_at) | 0.889683 | -0.151 | low density lipoprotein receptor |
| MAP3K12(205448_s_at) | 0.776679 | 0.0706 | mitogen-activated protein kinase kinase kinase 12 |
| MAP3K12(205447_s_at) | 0.910554 | 0.0564 | mitogen-activated protein kinase kinase kinase 12 |
| MAPK9(210570_x_at) | 0.591669 | 0.106 | mitogen-activated protein kinase 9 |
| MAPK9(203218_at) | 0.902636 | 0.0223 | mitogen-activated protein kinase 9 |
| MDM2(205385_at) | 0.092524 | -0.877 | MDM2 proto-oncogene |
| MDM2(211832_s_at) | 0.112749 | -0.595 | MDM2 proto-oncogene |
| MDM2(217542_at) | 0.178678 | 0.984 | MDM2 proto-oncogene |
| MDM2(205386_s_at) | 0.443303 | -0.253 | MDM2 proto-oncogene |
| MDM2(217373_x_at) | 0.578521 | -0.255 | MDM2 proto-oncogene |
| MEF2D(203004_s_at) | 0.056512 | 1.24 | myocyte enhancer factor 2D |
| MEF2D(203003_at) | 0.965125 | 0.0193 | myocyte enhancer factor 2D |
| MFN2(201155_s_at) | 0.424196 | 0.187 | mitofusin 2 |
| MFN2(216205_s_at) | 0.687231 | -0.0918 | mitofusin 2 |
| MMP2 | 0.457158 | -0.478 | matrix metallopeptidase 2 |
| MYC | 0.69315 | -0.112 | v-myc avian myelocytomatosis viral oncogene homolog |
| NABP1 | 0.537682 | -0.561 | nucleic acid binding protein 1 |
| NCOA3(209062_x_at) | 0.411752 | -0.239 | nuclear receptor coactivator 3 |
| NCOA3(209060_x_at) | 0.518008 | -0.128 | nuclear receptor coactivator 3 |
| NCOA3(211352_s_at) | 0.673792 | 0.115 | nuclear receptor coactivator 3 |
| NCOA3(207700_s_at) | 0.871173 | -0.0288 | nuclear receptor coactivator 3 |
| NCOA3(209061_at) | 0.891984 | 0.0265 | nuclear receptor coactivator 3 |
| NPAS3(220316_at) | 0.105099 | 0.954 | neuronal PAS domain protein 3 |
| NPAS3(222172_at) | 0.209795 | -0.695 | neuronal PAS domain protein 3 |
| NPAT(211585_at) | 0.290452 | -0.597 | nuclear protein, coactivator of histone transcription |
| NPAT(211584_s_at) | 0.42458 | 0.216 | nuclear protein, coactivator of histone transcription |
| NPAT(209798_at) | 0.945546 | -0.018 | nuclear protein, coactivator of histone transcription |
| PDLIM7(214266_s_at) | 0.112882 | 1.31 | PDZ and LIM domain 7 |
| PDLIM7(203369_x_at) | 0.197694 | -0.826 | PDZ and LIM domain 7 |
| PDLIM7(203370_s_at) | 0.71349 | 0.297 | PDZ and LIM domain 7 |
| PDLIM7(214121_x_at) | 0.819061 | -0.183 | PDZ and LIM domain 7 |
| PDLIM7(214122_at) | 0.986648 | 0.00925 | PDZ and LIM domain 7 |
| PHLPP1(212719_at) | 0.195399 | 0.363 | PH domain and leucine rich repeat protein phosphatase 1 |
| PHLPP1(210919_at) | 0.249548 | 1.15 | PH domain and leucine rich repeat protein phosphatase 1 |
| PKD2 | 0.477494 | -0.17 | polycystin 2, transient receptor potential cation channel |
| PKNOX1(204196_x_at) | 0.329817 | 0.307 | PBX/knotted 1 homeobox 1 |
| PKNOX1(221883_at) | 0.706924 | 0.104 | PBX/knotted 1 homeobox 1 |
| PKNOX1(204195_s_at) | 0.738078 | 0.274 | PBX/knotted 1 homeobox 1 |
| PKNOX1(54051_at) | 0.83736 | -0.0789 | PBX/knotted 1 homeobox 1 |
| PKNOX1(216004_s_at) | 0.894813 | -0.159 | PBX/knotted 1 homeobox 1 |
| PPP2R2A | 0.706181 | -0.063 | protein phosphatase 2 regulatory subunit Balpha |
| PTEN(204053_x_at) | 0.153471 | 0.295 | phosphatase and tensin homolog |
| PTEN(211711_s_at) | 0.312692 | 0.193 | phosphatase and tensin homolog |
| PTEN(204054_at) | 0.859223 | -0.0345 | phosphatase and tensin homolog |
| PTPRO(211600_at) | 0.023608 | 0.757 | protein tyrosine phosphatase, receptor type O |
| PTPRO(208121_s_at) | 0.713736 | 0.0986 | protein tyrosine phosphatase, receptor type O |
| RAD21(200607_s_at) | 0.157171 | -0.332 | RAD21 cohesin complex component |
| RAD21(200608_s_at) | 0.791253 | -0.0543 | RAD21 cohesin complex component |
| RB1(211540_s_at) | 0.442331 | 0.349 | RB transcriptional corepressor 1 |
| RB1(203132_at) | 0.505177 | 0.129 | RB transcriptional corepressor 1 |
| RBL1 | 0.422674 | 0.181 | RB transcriptional corepressor like 1 |
| RBL2(212331_at) | 0.20324 | 0.307 | RB transcriptional corepressor like 2 |
| RBL2(212332_at) | 0.364211 | 0.315 | RB transcriptional corepressor like 2 |
| RND3 | 0.662787 | -0.172 | Rho family GTPase 3 |
| RUNX1 | NA | NA |  |
| SIRPA(202895_s_at) | 0.31656 | -0.476 | signal regulatory protein alpha |
| SIRPA(202897_at) | 0.325415 | -0.785 | signal regulatory protein alpha |
| SIRPA(202896_s_at) | 0.888451 | -0.0609 | signal regulatory protein alpha |
| SMAD4(202527_s_at) | 0.055601 | 0.375 | SMAD family member 4 |
| SMAD4(202526_at) | 0.293851 | -0.268 | SMAD family member 4 |
| SMURF1(212666_at) | 0.416173 | 0.164 | SMAD specific E3 ubiquitin protein ligase 1 |
| SMURF1(212668_at) | 0.728526 | 0.141 | SMAD specific E3 ubiquitin protein ligase 1 |
| SMURF1(215458_s_at) | 0.755199 | 0.108 | SMAD specific E3 ubiquitin protein ligase 1 |
| SOCS6(206020_at) | 0.237853 | 0.562 | suppressor of cytokine signaling 6 |
| SOCS6(214462_at) | 0.342721 | 0.288 | suppressor of cytokine signaling 6 |
| STAT3(208992_s_at) | 0.576268 | 0.126 | signal transducer and activator of transcription 3 |
| STAT3(208991_at) | 0.708943 | 0.0836 | signal transducer and activator of transcription 3 |
| TBC1D2 | 0.462226 | -0.635 | TBC1 domain family member 2 |
| TCEAL1 | 0.340309 | 0.258 | transcription elongation factor A like 1 |
| TCF3(213730_x_at) | 0.105573 | -0.374 | transcription factor 3 |
| TCF3(209153_s_at) | 0.164129 | 0.303 | transcription factor 3 |
| TCF3(202648_at) | 0.268026 | -0.877 | transcription factor 3 |
| TCF3(213731_s_at) | 0.312167 | -0.395 | transcription factor 3 |
| TCF3(213809_x_at) | 0.324065 | -0.521 | transcription factor 3 |
| TCF3(209151_x_at) | 0.395433 | -0.542 | transcription factor 3 |
| TCF3(216645_at) | 0.407081 | -0.453 | transcription factor 3 |
| TCF3(213811_x_at) | 0.452397 | -0.166 | transcription factor 3 |
| TCF3(215260_s_at) | 0.453936 | 0.458 | transcription factor 3 |
| TCF3(210776_x_at) | 0.533938 | 0.217 | transcription factor 3 |
| TCF3(209152_s_at) | 0.589516 | -0.292 | transcription factor 3 |
| TCF3(213732_at) | 0.913934 | 0.0414 | transcription factor 3 |
| TCF3(216647_at) | 0.947161 | 0.0372 | transcription factor 3 |
| TGFBR2(208944_at) | 0.030343 | 0.613 | transforming growth factor beta receptor 2 |
| TGFBR2(207334_s_at) | 0.905338 | 0.112 | transforming growth factor beta receptor 2 |
| TIMP3(201150_s_at) | 0.165161 | -0.788 | TIMP metallopeptidase inhibitor 3 |
| TIMP3(201148_s_at) | 0.645268 | -0.112 | TIMP metallopeptidase inhibitor 3 |
| TIMP3(201147_s_at) | 0.680208 | 0.233 | TIMP metallopeptidase inhibitor 3 |
| TIMP3(201149_s_at) | 0.971016 | -0.00979 | TIMP metallopeptidase inhibitor 3 |
| TLR7 | 0.207384 | 0.753 | toll like receptor 7 |
| TNF | 0.200215 | 0.484 | tumor necrosis factor |
| TNFSF12 | NA | NA |  |
| TP53COR1 | NA | NA |  |
| TP53INP1 | NA | NA |  |
| TRIM8 | 0.647564 | 0.0979 | tripartite motif containing 8 |
| UBE2C | 0.136557 | -0.32 | ubiquitin conjugating enzyme E2 C |
| VEGFA(211527_x_at) | 0.526959 | -0.312 | vascular endothelial growth factor A |
| VEGFA(210512_s_at) | 0.637342 | 0.566 | vascular endothelial growth factor A |
| VEGFA(212171_x_at) | 0.670405 | 0.196 | vascular endothelial growth factor A |
| VEGFA(210513_s_at) | 0.765493 | 0.0725 | vascular endothelial growth factor A |
| VLDLR | 0.410151 | -0.273 | very low density lipoprotein receptor |
| WEE1(212533_at) | 0.26683 | 0.418 | WEE1 G2 checkpoint kinase |
| WEE1(215711_s_at) | 0.522852 | 0.262 | WEE1 G2 checkpoint kinase |
| YES1(202933_s_at) | 0.123962 | 0.71 | YES proto-oncogene 1, Src family tyrosine kinase |
| YES1(202932_at) | 0.812457 | 0.0803 | YES proto-oncogene 1, Src family tyrosine kinase |
| YES1(210917_at) | 0.884041 | 0.124 | YES proto-oncogene 1, Src family tyrosine kinase |
| ZBTB4 | NA | NA |  |
| ZFYVE9(204893_s_at) | 0.401992 | 0.392 | zinc finger FYVE-type containing 9 |
| ZFYVE9(208446_s_at) | 0.458351 | -0.275 | zinc finger FYVE-type containing 9 |
| ZNFX1 | NA | NA |  |

Gene expression for miR-17-5p targets in CD4+ T-cells of people living with HIV compared with uninfected controls; Gene expression data obtained through Gene Expression Omnibus [22], dataset: GSE6740 [23]; NA=not available

**Table S8 Expression of target genes of miR-199a-3p in CD4+ T-cells**

| Gene (probeset) | P.Value | logFC | Gene.title |
| --- | --- | --- | --- |
| APOE(212884_x_at) | 0.979483 | 0.011 | apolipoprotein E |
| APOE(203381_s_at) | 0.656696 | -0.549 | apolipoprotein E |
| APOE(203382_s_at) | 0.885131 | 0.112 | apolipoprotein E |
| CAV2(203323_at) | 0.198715 | 0.56 | caveolin 2 |
| CAV2(213426_s_at) | 0.720967 | 0.146 | caveolin 2 |
| CAV2(203324_s_at) | 0.073812 | 2.23 | caveolin 2 |
| CD44(204489_s_at) | 0.131544 | 0.267 | CD44 molecule (Indian blood group) |
| CD44(204490_s_at) | 0.015707 | 0.556 | CD44 molecule (Indian blood group) |
| CD44(216056_at) | 0.530252 | 0.467 | CD44 molecule (Indian blood group) |
| CD44(209835_x_at) | 0.04528 | 0.621 | CD44 molecule (Indian blood group) |
| CD44(217523_at) | 0.650167 | -0.137 | CD44 molecule (Indian blood group) |
| CD44(212014_x_at) | 0.057988 | 0.615 | CD44 molecule (Indian blood group) |
| CD44(210916_s_at) | 0.041785 | 0.517 | CD44 molecule (Indian blood group) |
| CD44(212063_at) | 0.274953 | 0.178 | CD44 molecule (Indian blood group) |
| CDK7 | 0.243061 | 0.228 | cyclin dependent kinase 7 |
| DNAJA4 | 0.586314 | -0.124 | DnaJ heat shock protein family (Hsp40) member A4 |
| FLT1(204406_at) | 0.659951 | -0.37 | fms related tyrosine kinase 1 |
| FLT1(222033_s_at) | 0.836065 | -0.131 | fms related tyrosine kinase 1 |
| FLT1(210287_s_at) | 0.146248 | -0.994 | fms related tyrosine kinase 1 |
| FOXA2(210103_s_at) | 0.098257 | -0.564 | forkhead box A2 |
| FOXA2(40284_at) | 0.391342 | -0.436 | forkhead box A2 |
| FOXA2(214312_at) | 0.779701 | 0.0708 | forkhead box A2 |
| FUT4(209892_at) | 0.409441 | 0.233 | fucosyltransferase 4 |
| FUT4(209893_s_at) | 0.951644 | -0.0283 | fucosyltransferase 4 |
| HGF(209960_at) | 0.803119 | -0.194 | hepatocyte growth factor |
| HGF(210998_s_at) | 0.47223 | -0.355 | hepatocyte growth factor |
| HGF(209961_s_at) | 0.666438 | 0.14 | hepatocyte growth factor |
| HGF(210997_at) | 0.802685 | -0.132 | hepatocyte growth factor |
| HGF(210755_at) | 0.59138 | -0.167 | hepatocyte growth factor |
| IGF1(209541_at) | 0.638536 | -0.162 | insulin like growth factor 1 |
| IGF1(209542_x_at) | 0.229684 | -0.702 | insulin like growth factor 1 |
| IGF1(211577_s_at) | 0.585006 | 0.394 | insulin like growth factor 1 |
| IGF1(209540_at) | 0.816258 | 0.154 | insulin like growth factor 1 |
| ITGA3 | 0.840478 | 0.0951 | integrin subunit alpha 3 |
| KDR | 0.739195 | 0.229 | kinase insert domain receptor |
| MAPK1(208351_s_at) | 0.664037 | -0.0989 | mitogen-activated protein kinase 1 |
| MAPK1(212271_at) | 0.585756 | -0.102 | mitogen-activated protein kinase 1 |
| MET(211599_x_at) | 0.751086 | 0.102 | MET proto-oncogene, receptor tyrosine kinase |
| MET(203510_at) | 0.258596 | -0.474 | MET proto-oncogene, receptor tyrosine kinase |
| MET(213807_x_at) | 0.437287 | 0.363 | MET proto-oncogene, receptor tyrosine kinase |
| MET(213816_s_at) | 0.965624 | 0.0174 | MET proto-oncogene, receptor tyrosine kinase |
| MTOR(202288_at) | 0.681853 | -0.104 | mechanistic target of rapamycin |
| MTOR(215381_at) | 0.170979 | -0.975 | mechanistic target of rapamycin |
| PAK4(215326_at) | 0.483664 | -0.263 | p21 (RAC1) activated kinase 4 |
| PAK4(33814_at) | 0.682843 | -0.16 | p21 (RAC1) activated kinase 4 |
| PAK4(203154_s_at) | 0.886208 | -0.0457 | p21 (RAC1) activated kinase 4 |
| PTGS2 | 0.467962 | 0.616 | prostaglandin-endoperoxide synthase 2 |
| SMARCA2(217707_x_at) | 0.739117 | -0.0644 | SWI/SNF related, matrix associated, actin dependent regulator of chromatin, subfamily a, member 2 |
| SMARCA2(212258_s_at) | 0.103923 | -0.663 | SWI/SNF related, matrix associated, actin dependent regulator of chromatin, subfamily a, member 2 |
| SMARCA2(206542_s_at) | 0.672329 | -0.0904 | SWI/SNF related, matrix associated, actin dependent regulator of chromatin, subfamily a, member 2 |
| SMARCA2(212257_s_at) | 0.845716 | -0.0494 | SWI/SNF related, matrix associated, actin dependent regulator of chromatin, subfamily a, member 2 |
| SMARCA2(206543_at) | 0.85262 | -0.171 | SWI/SNF related, matrix associated, actin dependent regulator of chromatin, subfamily a, member 2 |
| SMARCA2(206544_x_at) | 0.997326 | 0.000696 | SWI/SNF related, matrix associated, actin dependent regulator of chromatin, subfamily a, member 2 |
| STK11(41657_at) | 0.276909 | 0.311 | serine/threonine kinase 11 |
| STK11(204292_x_at) | 0.689037 | -0.259 | serine/threonine kinase 11 |
| TFAM(203177_x_at) | 0.568207 | 0.111 | transcription factor A, mitochondrial |
| TFAM(208541_x_at) | 0.183778 | -0.471 | transcription factor A, mitochondrial |
| TFAM(203176_s_at) | 0.633389 | -0.101 | transcription factor A, mitochondrial |
| VEGFA(211527_x_at) | 0.526959 | -0.312 | vascular endothelial growth factor A |
| VEGFA(210512_s_at) | 0.637342 | 0.566 | vascular endothelial growth factor A |
| VEGFA(212171_x_at) | 0.670405 | 0.196 | vascular endothelial growth factor A |
| VEGFA(210513_s_at) | 0.765493 | 0.0725 | vascular endothelial growth factor A |
| YAP1 | 0.594829 | -0.344 | Yes associated protein 1 |
| ZHX1 | NA | NA |  |

Gene expression for miR-199a-3p targets in CD4+ T-cells of people living with HIV compared with uninfected controls; Gene expression data obtained through Gene Expression Omnibus [22], dataset: GSE6740 [23]; NA=not available

**Table S9 Expression of target genes of miR-200c-3p in CD4+ T-cells**

| Gene (probeset | P.Value | logFC | Gene.title |
| --- | --- | --- | --- |
| ACVR2B | 0.508452 | -0.168 | activin A receptor type 2B |
| ADAM12(215613_at) | 0.42314 | -0.58 | ADAM metallopeptidase domain 12 |
| ADAM12(213790_at) | 0.244079 | 0.602 | ADAM metallopeptidase domain 12 |
| ADAM12(202952_s_at) | 0.816289 | 0.175 | ADAM metallopeptidase domain 12 |
| ADAM12(204943_at) | 0.659025 | -0.232 | ADAM metallopeptidase domain 12 |
| ATRX(208859_s_at) | 0.483634 | -0.181 | ATRX, chromatin remodeler |
| ATRX(208860_s_at) | 0.465966 | -0.152 | ATRX, chromatin remodeler |
| ATRX(208861_s_at) | 0.617461 | 0.0875 | ATRX, chromatin remodeler |
| ATRX(211022_s_at) | 0.46445 | -0.263 | ATRX, chromatin remodeler |
| BAP1 | 0.895243 | -0.0339 | BRCA1 associated protein 1 |
| BCL2(203684_s_at) | 0.113466 | -1.14 | BCL2, apoptosis regulator |
| BCL2(203685_at) | 0.23601 | -0.264 | BCL2, apoptosis regulator |
| BCL2(207004_at) | 0.499308 | 0.182 | BCL2, apoptosis regulator |
| BCL2(207005_s_at) | 0.789358 | -0.0825 | BCL2, apoptosis regulator |
| BMI1 | NA | NA |  |
| BRD7 | 0.990318 | -0.00237 | bromodomain containing 7 |
| BTC | 0.937211 | 0.0575 | betacellulin |
| CCNE2(211814_s_at) | 0.585809 | -0.298 | cyclin E2 |
| CCNE2(205034_at) | 0.121467 | -0.823 | cyclin E2 |
| CDH11(207172_s_at) | 0.240432 | -0.411 | cadherin 11 |
| CDH11(207173_x_at) | 0.679081 | -0.238 | cadherin 11 |
| CDK2(204252_at) | 0.875711 | 0.0297 | cyclin dependent kinase 2 |
| CDK2(211803_at) | 0.147187 | 0.851 | cyclin dependent kinase 2 |
| CDK2(211804_s_at) | 0.652141 | -0.287 | cyclin dependent kinase 2 |
| CFL2 | NA | NA |  |
| CRKL(206184_at) | 0.282715 | -0.246 | CRK like proto-oncogene, adaptor protein |
| CRKL(212180_at) | 0.195192 | 0.236 | CRK like proto-oncogene, adaptor protein |
| DLC1(220512_at) | 0.614868 | 0.28 | DLC1 Rho GTPase activating protein |
| DLC1(220511_s_at) | 0.458006 | 0.256 | DLC1 Rho GTPase activating protein |
| DLC1(210762_s_at) | 0.83661 | 0.118 | DLC1 Rho GTPase activating protein |
| DNAJC3 | 0.606406 | -0.411 | DnaJ heat shock protein family (Hsp40) member C3 |
| DNMT3A | 0.842753 | -0.0591 | DNA methyltransferase 3 alpha |
| DNMT3B | 0.465171 | 0.228 | DNA methyltransferase 3 beta |
| DUSP1(201041_s_at) | 0.746484 | -0.0894 | dual specificity phosphatase 1 |
| DUSP1(201044_x_at) | 0.612814 | 0.333 | dual specificity phosphatase 1 |
| E2F3(203693_s_at) | 0.574569 | -0.136 | E2F transcription factor 3 |
| E2F3(203692_s_at) | 0.927782 | 0.0146 | E2F transcription factor 3 |
| EDNRA(204464_s_at) | 0.99035 | 0.00572 | endothelin receptor type A |
| EDNRA(204463_s_at) | 0.799705 | 0.174 | endothelin receptor type A |
| EDNRA(216235_s_at) | 0.798334 | -0.24 | endothelin receptor type A |
| EFNA1 | 0.111347 | 0.501 | ephrin A1 |
| ELMO2(220363_s_at) | 0.378198 | 0.516 | engulfment and cell motility 2 |
| ELMO2(221528_s_at) | 0.668062 | 0.0909 | engulfment and cell motility 2 |
| ELMO2(55692_at) | 0.996949 | 0.000686 | engulfment and cell motility 2 |
| ERBIN | 0.340497 | 0.2 | erbb2 interacting protein |
| ERRFI1 | NA | NA |  |
| ETS1 | 0.242118 | 0.27 | ETS proto-oncogene 1, transcription factor |
| FBLN5 | 0.195569 | 0.926 | fibulin 5 |
| FLT1(204406_at) | 0.659951 | -0.37 | fms related tyrosine kinase 1 |
| FLT1(222033_s_at) | 0.836065 | -0.131 | fms related tyrosine kinase 1 |
| FLT1(210287_s_at) | 0.146248 | -0.994 | fms related tyrosine kinase 1 |
| FN1(214701_s_at) | 0.318119 | 0.254 | fibronectin 1 |
| FN1(214702_at) | 0.607671 | 0.358 | fibronectin 1 |
| FN1(211719_x_at) | 0.150497 | -0.569 | fibronectin 1 |
| FN1(212464_s_at) | 0.511035 | -0.287 | fibronectin 1 |
| FN1(216442_x_at) | 0.007128 | 0.983 | fibronectin 1 |
| FN1(210495_x_at) | 0.927949 | 0.0259 | fibronectin 1 |
| FOXO1(202724_s_at) | 0.0428 | 0.412 | forkhead box O1 |
| FOXO1(202723_s_at) | 0.151537 | 0.708 | forkhead box O1 |
| GATA4 | 0.533354 | 0.284 | GATA binding protein 4 |
| GEMIN2(210779_x_at) | 0.34762 | -0.251 | gem nuclear organelle associated protein 2 |
| GEMIN2(205063_at) | 0.167555 | 0.322 | gem nuclear organelle associated protein 2 |
| GEMIN2(211115_x_at) | 0.869534 | 0.0295 | gem nuclear organelle associated protein 2 |
| GEMIN2(211114_x_at) | 0.521739 | -0.113 | gem nuclear organelle associated protein 2 |
| HFE(211331_x_at) | 0.31412 | -0.485 | hemochromatosis |
| HFE(211863_x_at) | 0.069962 | -0.825 | hemochromatosis |
| HFE(206087_x_at) | 0.141441 | -0.451 | hemochromatosis |
| HFE(211327_x_at) | 0.772947 | 0.0961 | hemochromatosis |
| HFE(211866_x_at) | 0.25642 | -0.498 | hemochromatosis |
| HFE(211326_x_at) | 0.145682 | -0.576 | hemochromatosis |
| HFE(211329_x_at) | 0.993017 | -0.00293 | hemochromatosis |
| HFE(210864_x_at) | 0.48292 | -0.272 | hemochromatosis |
| HFE(214647_s_at) | 0.078658 | -0.68 | hemochromatosis |
| HFE(211330_s_at) | 0.202607 | -0.444 | hemochromatosis |
| HFE(206086_x_at) | 0.020241 | -0.672 | hemochromatosis |
| HFE(211332_x_at) | 0.720697 | -0.0644 | hemochromatosis |
| HFE(211328_x_at) | 0.86819 | -0.0422 | hemochromatosis |
| HOXB5(205601_s_at) | 0.99948 | -0.00024 | homeobox B5 |
| HOXB5(205600_x_at) | 0.446447 | -0.213 | homeobox B5 |
| IKBKB(211027_s_at) | 0.914336 | -0.0656 | inhibitor of kappa light polypeptide gene enhancer in B-cells, kinase beta |
| IKBKB(209342_s_at) | 0.609529 | 0.12 | inhibitor of kappa light polypeptide gene enhancer in B-cells, kinase beta |
| IKBKB(209341_s_at) | 0.859612 | -0.0352 | inhibitor of kappa light polypeptide gene enhancer in B-cells, kinase beta |
| JAZF1 | NA | NA |  |
| KDR | 0.739195 | 0.229 | kinase insert domain receptor |
| KLF11 | 0.079893 | 0.546 | Kruppel like factor 11 |
| KLF9(203541_s_at) | 0.566658 | 0.293 | Kruppel like factor 9 |
| KLF9(203542_s_at) | 0.203464 | 0.366 | Kruppel like factor 9 |
| KLF9(203543_s_at) | 0.015325 | 0.658 | Kruppel like factor 9 |
| KLHL20(210635_s_at) | 0.226568 | 0.202 | kelch like family member 20 |
| KLHL20(210634_at) | 0.956835 | 0.0114 | kelch like family member 20 |
| KLHL20(204177_s_at) | 0.937897 | 0.0122 | kelch like family member 20 |
| KLHL20(204176_at) | 0.685857 | 0.14 | kelch like family member 20 |
| KRAS(204010_s_at) | 0.417655 | -0.261 | KRAS proto-oncogene, GTPase |
| KRAS(214352_s_at) | 0.098215 | -0.29 | KRAS proto-oncogene, GTPase |
| KRAS(204009_s_at) | 0.517438 | 0.11 | KRAS proto-oncogene, GTPase |
| LEPR | NA | NA |  |
| LPAR1(204038_s_at) | 0.336895 | 0.298 | lysophosphatidic acid receptor 1 |
| LPAR1(204037_at) | 0.336212 | 0.79 | lysophosphatidic acid receptor 1 |
| LPAR1(204036_at) | 0.541265 | 0.452 | lysophosphatidic acid receptor 1 |
| MALAT1 | NA | NA |  |
| MSN | 0.673962 | 0.0604 | moesin |
| MYB(215152_at) | 0.186838 | -1.12 | MYB proto-oncogene, transcription factor |
| MYB(204798_at) | 0.360791 | -0.216 | MYB proto-oncogene, transcription factor |
| MYLK | 0.25604 | 1.16 | myosin light chain kinase |
| NCAM1(212843_at) | 0.668685 | -0.211 | neural cell adhesion molecule 1 |
| NCAM1(209968_s_at) | 0.589644 | 0.395 | neural cell adhesion molecule 1 |
| NCAM1(214952_at) | 0.217551 | 0.401 | neural cell adhesion molecule 1 |
| NCAM1(217359_s_at) | 0.832058 | -0.0631 | neural cell adhesion molecule 1 |
| NOS3 | 0.366696 | -0.462 | nitric oxide synthase 3 |
| NOTCH1 | 0.682139 | 0.198 | notch 1 |
| NTF3 | 0.614308 | -0.257 | neurotrophin 3 |
| NTRK2(221795_at) | 0.788708 | -0.201 | neurotrophic receptor tyrosine kinase 2 |
| NTRK2(207152_at) | 0.785024 | 0.0953 | neurotrophic receptor tyrosine kinase 2 |
| NTRK2(214680_at) | 0.360667 | 0.516 | neurotrophic receptor tyrosine kinase 2 |
| NTRK2(221796_at) | 0.31396 | 0.622 | neurotrophic receptor tyrosine kinase 2 |
| PDCD10 | 0.0683 | -0.388 | programmed cell death 10 |
| PIN1 | 0.831299 | -0.0766 | peptidylprolyl cis/trans isomerase, NIMA-interacting 1 |
| PMAIP1(204286_s_at) | 0.130774 | -0.444 | phorbol-12-myristate-13-acetate-induced protein 1 |
| PMAIP1(204285_s_at) | 0.054529 | -0.568 | phorbol-12-myristate-13-acetate-induced protein 1 |
| PRKCZ | 0.050491 | 0.474 | protein kinase C zeta |
| PTEN(204053_x_at) | 0.153471 | 0.295 | phosphatase and tensin homolog |
| PTEN(211711_s_at) | 0.312692 | 0.193 | phosphatase and tensin homolog |
| PTEN(204054_at) | 0.859223 | -0.0345 | phosphatase and tensin homolog |
| PTPN13 | 0.651698 | 0.242 | protein tyrosine phosphatase, non-receptor type 13 |
| PTPRD(213362_at) | 0.913796 | -0.0696 | protein tyrosine phosphatase, receptor type D |
| PTPRD(214043_at) | 0.616646 | -0.379 | protein tyrosine phosphatase, receptor type D |
| PTPRD(205712_at) | 0.556087 | -0.304 | protein tyrosine phosphatase, receptor type D |
| RASSF2 | 0.261043 | 0.266 | Ras association domain family member 2 |
| RCOR3 | 0.349869 | -0.173 | REST corepressor 3 |
| RHOA | 0.916501 | -0.0168 | ras homolog family member A |
| RIN2 | 0.751414 | -0.175 | Ras and Rab interactor 2 |
| RND3 | 0.662787 | -0.172 | Rho family GTPase 3 |
| RNF2 | 0.763976 | 0.122 | ring finger protein 2 |
| ROCK2(211504_x_at) | 0.454115 | 0.211 | Rho associated coiled-coil containing protein kinase 2 |
| ROCK2(202762_at) | 0.082412 | 0.49 | Rho associated coiled-coil containing protein kinase 2 |
| RPS6KB1(211578_s_at) | 0.767364 | -0.152 | ribosomal protein S6 kinase B1 |
| RPS6KB1(204171_at) | 0.58181 | -0.193 | ribosomal protein S6 kinase B1 |
| SEC23A(212887_at) | 0.58379 | 0.108 | Sec23 homolog A, coat complex II component |
| SEC23A(204344_s_at) | 0.744733 | 0.234 | Sec23 homolog A, coat complex II component |
| SEPTIN7 | NA | NA |  |
| SH3PXD2A(213252_at) | 0.460502 | 0.271 | SH3 and PX domains 2A |
| SH3PXD2A(207661_s_at) | 0.181046 | -0.678 | SH3 and PX domains 2A |
| SHC1(201469_s_at) | 0.77385 | -0.108 | SHC adaptor protein 1 |
| SHC1(214853_s_at) | 0.337913 | 0.202 | SHC adaptor protein 1 |
| SIRT1 | 0.372832 | 0.183 | sirtuin 1 |
| SLC1A2(217055_x_at) | 0.927203 | 0.0349 | solute carrier family 1 member 2 |
| SLC1A2(217037_at) | 0.055168 | 1.39 | solute carrier family 1 member 2 |
| SLC1A2(208389_s_at) | 0.70109 | 0.268 | solute carrier family 1 member 2 |
| SP1 | 0.077459 | -0.995 | Sp1 transcription factor |
| SUZ12 | 0.743503 | -0.0512 | SUZ12 polycomb repressive complex 2 subunit |
| TBK1 | 0.096185 | -0.336 | TANK binding kinase 1 |
| TCF7L1 | 0.714974 | 0.178 | transcription factor 7 like 1 |
| TIMP2 | 0.795507 | -0.107 | TIMP metallopeptidase inhibitor 2 |
| TUBB3(213476_x_at) | 0.318376 | -0.173 | tubulin beta 3 class III |
| TUBB3(202154_x_at) | 0.364334 | -0.156 | tubulin beta 3 class III |
| UBQLN1 | NA | NA |  |
| USP25 | 0.998392 | 0.000341 | ubiquitin specific peptidase 25 |
| VAC14(216501_at) | 0.142296 | -0.471 | Vac14, PIKFYVE complex component |
| VAC14(218169_at) | 0.022507 | 1.17 | Vac14, PIKFYVE complex component |
| VAC14(216407_at) | 0.144196 | -1.03 | Vac14, PIKFYVE complex component |
| VEGFA(211527_x_at) | 0.526959 | -0.312 | vascular endothelial growth factor A |
| VEGFA(210512_s_at) | 0.637342 | 0.566 | vascular endothelial growth factor A |
| VEGFA(212171_x_at) | 0.670405 | 0.196 | vascular endothelial growth factor A |
| VEGFA(210513_s_at) | 0.765493 | 0.0725 | vascular endothelial growth factor A |
| WDR37 | 0.193225 | 0.332 | WD repeat domain 37 |
| XIAP(206537_at) | 0.244661 | -1.01 | X-linked inhibitor of apoptosis |
| XIAP(206536_s_at) | 0.252692 | -0.505 | X-linked inhibitor of apoptosis |
| ZEB1(212758_s_at) | 0.473121 | 0.263 | zinc finger E-box binding homeobox 1 |
| ZEB1(212764_at) | 0.158054 | 0.287 | zinc finger E-box binding homeobox 1 |
| ZEB1(210875_s_at) | 0.983617 | -0.00714 | zinc finger E-box binding homeobox 1 |
| ZEB2 | 0.488359 | -0.565 | zinc finger E-box binding homeobox 2 |
| ZFPM1 | NA | NA |  |
| ZFPM2 | 0.44339 | 0.459 | zinc finger protein, FOG family member 2 |
| ZNF217 | 0.620413 | -0.11 | zinc finger protein 217 |

Gene expression for miR-200c-3p targets in CD4+ T-cells of people living with HIV compared with uninfected controls; Gene expression data obtained through Gene Expression Omnibus [22], dataset: GSE6740 [23]; NA=not available
